# Phylogenetic insights into the genetic legacies of Hungarian-speaking communities in the Carpathian Basin

**DOI:** 10.1038/s41598-024-61978-4

**Published:** 2024-05-20

**Authors:** Noémi Borbély, Dániel Dudás, Attila Tapasztó, Eszter Dudás-Boda, Veronika Csáky, Bea Szeifert, Balázs Gusztáv Mende, Balázs Egyed, Anna Szécsényi-Nagy, Horolma Pamjav

**Affiliations:** 1 Institute of Archaeogenomics, HUN-REN Research Centre for the Humanities, Tóth Kálmán utca 4, Budapest, 1097 Hungary; 2 Doctoral School of Biology, Institute of Biology, ELTE Eötvös Loránd University, Pázmány Péter sétány 1/C, Budapest, 1117 Hungary; 3 Department of Reference Sample Analysis, Institute of Forensic Genetics, Hungarian Institute for Forensic Sciences, Gyorskocsi u. 25, Budapest, 1027 Hungary; 4 Department of Genetics, ELTE Eötvös Loránd University, Pázmány Péter sétány 1/C, Budapest, 1117 Hungary

**Keywords:** Population genetics, Haplotypes

## Abstract

This study focuses on exploring the uniparental genetic lineages of Hungarian-speaking minorities residing in rural villages of Baranja (Croatia) and the Zobor region (Slovakia). We aimed to identify ancestral lineages by examining genetic markers distributed across the entire mitogenome and on the Y-chromosome. This allowed us to discern disparities in regional genetic structures within these communities. By integrating our newly acquired genetic data from a total of 168 participants with pre-existing Eurasian and ancient DNA datasets, our goal was to enrich the understanding of the genetic history trajectories of Carpathian Basin populations. Our findings suggest that while population-based analyses may not be sufficiently robust to detect fine-scale uniparental genetic patterns with the sample sizes at hand, phylogenetic analysis of well-characterized Y-chromosomal Short Tandem Repeat (STR) data and entire mitogenome sequences did uncover multiple lineage ties to far-flung regions and eras. While the predominant portions of both paternal and maternal DNA align with the East-Central European spectrum, rarer subhaplogroups and lineages have unveiled ancient ties to both prehistoric and historic populations spanning Europe and Eastern Eurasia. This research augments the expansive field of phylogenetics, offering critical perspectives on the genetic constitution and heritage of the communities in East-Central Europe.

## Introduction

The non-recombining region (NRY) of the human Y-chromosome has been extensively researched to elucidate the population history, origin, and migration patterns of human groups^1–3^. This specific region of the Y-chromosome remains unaffected by recombination and is passed down intact from one generation to the next, thereby providing insights into the paternal genetic lineages of studied populations. Human populations often share Y-chromosome lineages due to shared ancestries or historical paternal gene flow between groups. Similarly, mitochondrial DNA (mtDNA), another uniparentally inherited segment of the genome, has emerged as a significant tool in studying human evolution and population history. Human mitochondrial frequency-based methods leverage the frequency distribution of mtDNA haplotypes or haplogroups within populations to infer evolutionary and demographic patterns^4–7^, while whole mitochondrial sequence-based phylogeny is a valuable tool for reconstructing the evolutionary relationships among human populations^8^. Both Y-chromosomal and mitochondrial markers contribute to our understanding of human population genetics, migration history and forensic identification^2,9,10^.

Many peoples migrating from the east found a new home in the Carpathian Basin in the historical era, such as Sarmatians, Huns, Avars, conquering Hungarians and Cumanians. The Hungarian conqueror groups entered the Carpathian Basin at the end of the ninth century (in 895 AD) where they established their power center integrating the local populations^11^. They arrived from their previous settlement areas east of the Carpathian Basin. Archaeologically and historically, we consider them as the ancestors of the Hungarian people, likely forming a complex community organized on a tribal federation basis.

When the genetic profiles of contemporary populations are contrasted with those of ancient groups, it aids in understanding the roots of present-day genetic configurations. Recently, the number of ancient DNA (aDNA) results based on both uniparental and whole genome data has increased, which form a robust genetic foundation for comparing ancient and contemporary populations in the Carpathian Basin^7,12–20^.

Genetic data focusing on uniparental markers—specifically Y-STR (Y-chromosomal short tandem repeat) and Y-SNP (Y-chromosomal single nucleotide polymorphism)—from modern Hungarian-speaking populations are available in the literature^21–27^. Based on Y-chromosomal data, the contemporary Hungarian males possess few identical genetic markers to tenth–eleventh century (conqueror) Hungarians in the Carpathian Basin as well as to the recent Central/Inner Asian populations and populations in the Ural and Caucasus Mountains^12,16,23,26,28–31^.

Based on whole genome sequencing of ancient samples, the Hungarian conquerors may have had multiple components, some originating from the Ural region, others from the Central Eurasian steppe and the Volga region. The genomic results largely correspond with earlier uniparental marker studies^15,31^.

Some mtDNA data have also been published from the Hungarian conquerors and contemporary Hungarian speakers^7,12,27,32,33^. These mtDNA studies of early and present-day Hungarians revealed a diverse composition of maternal haplogroups with ca 25–30% Easter-Eurasian components in the ancient datasets, with marking dilution up to present day^12,32–34^. These data represent the influence of eastern migration into Central Europe, which is today the most strongly detectable in the Hungarian speaking Székely population in Romania. In addition to addressing the influence of foreign immigrants, it’s crucial to highlight the significant role played by the local populace in shaping the medieval Hungarian nation, as noted by Maár et al.^7^.

**Figure 1:**
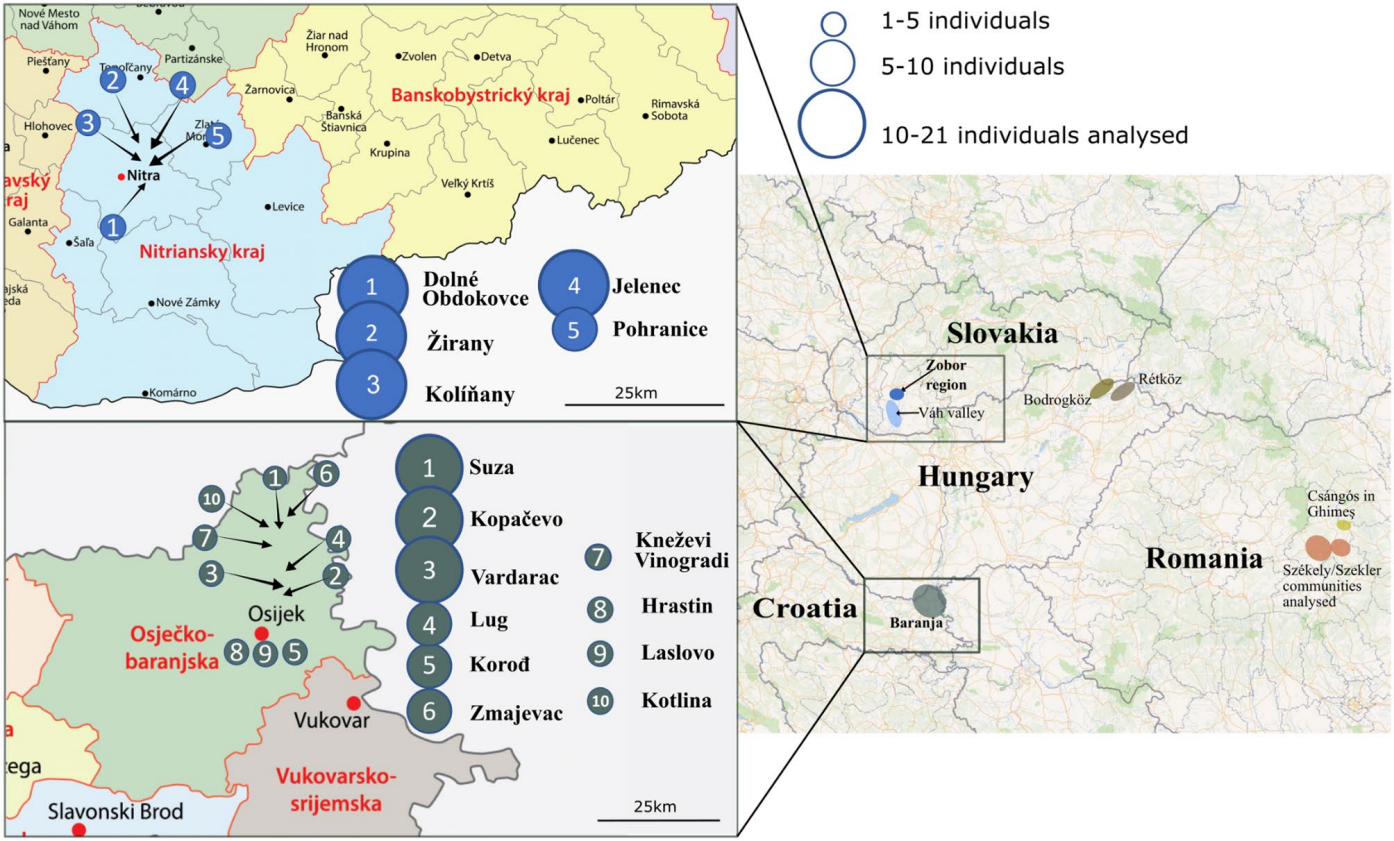
Map of sample collection. The geographical origin of 92 and 81 non-related Hungarian sample donors from Baranja, Croatia and Zobor region, Slovakia. Number of sampled individuals is proportional to the circle sizes on the maps, see the exact numbers for all villages in Supplementary Table S1. The left side shows the exact location of the sampled settlements within Slovakia and Croatia, and the right side shows the whole Carpathian Basin and its surroundings, with the previously published Hungarian-speaking groups^26,27,35,36^. Maps were taken from “*OpenSreetMap” (2023)*^37^ and *2023 worldatlas.com*^38,39^ and edited with Inkscape.

In this study, we investigate the uniparental gene pool of two Hungarian-speaking minority populations residing in Baranja, Croatia, and the Zobor region in Slovakia. We investigated the Northern Croatia area of Baranja (or Drávaszög in Hungarian), located between Danube and Drava rivers, close to Hungarian border, whereas the other sampling territory was the Zobor region (Zoboralja in Hungarian and Podzoborská oblasť, or Podzoborie in Slovakian) that is a historical landscape unit in Slovakia, which lies on the southern spurs of the Tribeč mountain range northeast of Nitra, the mountain chain between Zobor and Žibrica (Fig. 1). It originally included 13 villages founded in the Árpádian era (eleventh–thirteenth centuries). The samples were collected from the settlements at hillfoots of the Tribeč mountain’s eastern side. The common feature of the two regions is that Hungarians have been living there for approximately 1000 years in relative stability. See Supplementary Information Sect. 1 for a more detailed ethnohistorical description of the investigated regions.

Our primary objectives include genetic database construction and tracing the genetic lineage compositions of these contemporary populations back to approximately a century ago. This was achieved by identifying elderly individuals living in isolated villages and meticulously documenting their genealogies. Our goal is to detect uniparental lineages in these populations, observe their potential regional characteristics and compare them with archaeogenetic data. Furthermore, we aim to examine regional genetic structure disparities within the Hungarian-speaking population, as most genetic results to date have not been attributed to specific microregions of the Carpathian Basin but originated from random or clinical sampling of urban populations. Since maternal lineages from discrete present-day communities of Hungary have not yet been studied but are well-documented among the tenth–twelfth centuries Hungarians^7^, this approach facilitates comparative analyses and enhances our understanding of historical population processes.

## Results

We collected samples from 92 unrelated individuals from Baranja (81 males), Croatia and 81 from the Zobor region, Slovakia, with 40 of them being men (Fig. 1). Most of these individuals hailed from various villages predominantly inhabited by Hungarian-speaking minorities in both countries (as illustrated in Fig. 1*,* indicating the distribution of samples). Detailed ancestral documentation spanning two generations was available for these individuals, revealing that the majority of their ancestors were born in the same region of Slovakia/Croatia and self-identified as Hungarians.

We obtained novel genetic data consisting of 168 newly sequenced whole mitochondrial genomes and 23 Y-STR haplotypes widely used in forensic and population genetic studies and over 40 haplogroup defining Y-SNP profiles from 121 males.

### Y-chromosome diversity

To research the genetic variation within the Hungarian-speakers, we employed evolutionarily stable binary markers (SNPs) to define the haplogroup of each Y-chromosome. Subsequently, we examined the Y-STR variation of the groups, and specific phylogenetic analyses within eight selected haplogroups.

The Y haplogroup frequencies of the two populations are presented in Table 1 and on Fig. S1. Furthermore, the haplogroups’ origins and current distribution peaks, and ISOGG 2019–2020 names of the haplogroups can be found in Supplementary Table S3. The most frequent haplogroups in the Zobor region population were R1a-Z280 (32.5%), R1a-M458 (25%), R1b-P312 (15.00%), and G2a-L156 (7.5%). In the case of the Baranja males, the most frequent haplogroups were I2a-P37 (21.95%), R1a-Z280 (17.07%). The overall pattern of Y-chromosomal haplogroup distributions in the two studied populations were similar, but haplogroups R1a-Z93, N1c-M46, C2-M217, J2b-M12 appeared only in the Baranja population (Table 1), where G2a-L156 and R1b-M343/P25 (L23) were observed more frequently. We focused on the genetic history of these specific haplogroups (G2a-L156, R1b-L23) beside the R1a-Z280 and I2a-P37, as they have been previously shown to represent phylogeographically relevant structures^25,26^.

**Table 1:** Frequencies of the Y-chromosomal haplogroups from Baranja and Zobor region.

Haplogroup frequencies in Baranja, Croatia			Haplogroup frequencies in Zobor region, Slovakia			2019–2020 ISOGG nomenclature
Haplogroup	Sample number	Frequency (%)	Haplogroup	Sample number	Frequency (%)	Haplogroups
C-M216	1	1.22	C-M216	0	0	C (M217: C2)
E1b1b-M78	6	7.32	E1b1b-M78	1	2.5	E1b1b1a1
E1b1-M123	1	1.22	E1b1-M123	0	0	E1b1b1b2a1
G2a-L156	3	3.66	G2a-L156	3	7.5	G2 (P15: G2a)
I1-M253	8	9.76	I1-M253	0	0	I1
I2a-P37	18	21.95	I2a-P37	4	10.0	I2a1a
I2b-M223	1	1.22	I2b-M223	0	0	I2a1b1
J2b-M12	2	2.44	J2b-M12	0	0	J2b
L-M11	1	1.22	L-M11	0	0	L
N-VL29	1	1.22	N-VL29	0	0	N1a1a1a1a1a
R1a-M458	4	4.88	R1a-M458	10	25.0	R1a1a1b1a1a
R1a-Z280	14	17.07	R1a-Z280	13	32.5	R1a1a1b1a2
R1a-Z93	2	2.44	R1a-Z93	0	0	R1a1a1b2
R1b-M343*	1	1.22	R1b-M343*	0	0	R1b
R1b-M412	1	1.22	R1b-M412	1	2.5	R1b1a1b1a
R1b-P25*	8	9.76	R1b-P25*	1	2.5	L23: R1b1a1b1
R1b-P312	5	6.10	R1b-P312	6	15.0	R1b1a1b1a1a2
R1b-U106	4	4.88	R1b-U106	0	0	R1b1a1b1a1a1
T-M70	1	1.22	T-M70	0	0	T1a
R1a-M198*	0	0	R1a-M198*	1	2.5	R1a1a

The Baranja group exhibited haplotype and haplogroup diversities of 0.99938 and 0.90586, respectively. In contrast, the Zobor region displayed lower values, with 0.98974 for haplotype diversity and 0.81154 for haplogroup diversity. The Y-STR and Y-SNP outcomes for the 40 samples from the Zobor region (Slovakia) and the 81 samples from Baranja (Croatia) are detailed in Supplementary Table S3 and S6. The diminished diversity observed in the Zobor region might be attributed to the smaller sample size. However, this reduced diversity is still more pronounced (0.812 haplogroup diversity) than what was observed in the Váh valley group, presented by *n* = 48 Y-STR haplotypes^26^. On the other hand, bottleneck effect or drift is also likely in small, isolated populations, which may cause substructures in the Carpathian Basin.

### Paternal genetic structure of the two populations

The Y haplogroup frequency data were calculated incorporating reference populations and used for a PCA plot (Supplementary Table S7 and Fig. S2). The location of the studied populations on the PCA plot is roughly consistent with the geographical distances between them. Populations from the same geographic region were clustered together and Hungarian populations overlapped with the surrounding Slavic populations, and the Zobor region shows more connections to northern, northeastern populations. The resulted pattern with slight shift of the Zobor region sample set from the Baranja group is primarily due to the relatively high I2a, I1 and E1b1 haplogroup frequencies in Baranja populations. Further differences may be due to the preponderance (25%) of R1a-M458 in the Zobor region population, which is common among Western Slavs, and the absence of the R1b subgroup (U106), which is common in Western Europe. Interestingly, occurrence of J is relatively small in the Baranja population compared to other Hungarian groups and the Székelys, and is absent in the Zobor region group. The previously detected Q at the Székelys is also missing in the current two populations.

Pairwise F_ST_ distances and *p* values for 41 populations, including Baranja, Zobor region, and other Eurasian populations from published sources were calculated as shown in Supplementary Table S8 and presented in a heatmap plot with clustering (Fig. S3). The Zobor region shows significant genetic distance from almost every other group (*p* < 0.05), whereas the Baranja group is in non-significant distance from the pooled population of Hungary, the Székelys, Moldovans and Slovenians. While small sample sizes limit the scope of definitive conclusions, the clustering method groups populations with high genetic affinities to one another. Eastern Europeans and Hungarians from Hungary, the Baranja, and Zobor regions form one cohesive cluster. In contrast, populations with rather Southeastern European characteristics, including the Székelys and Csángós, constitute a distinct cluster (Supplementary Table S8).

**Figure 2:**
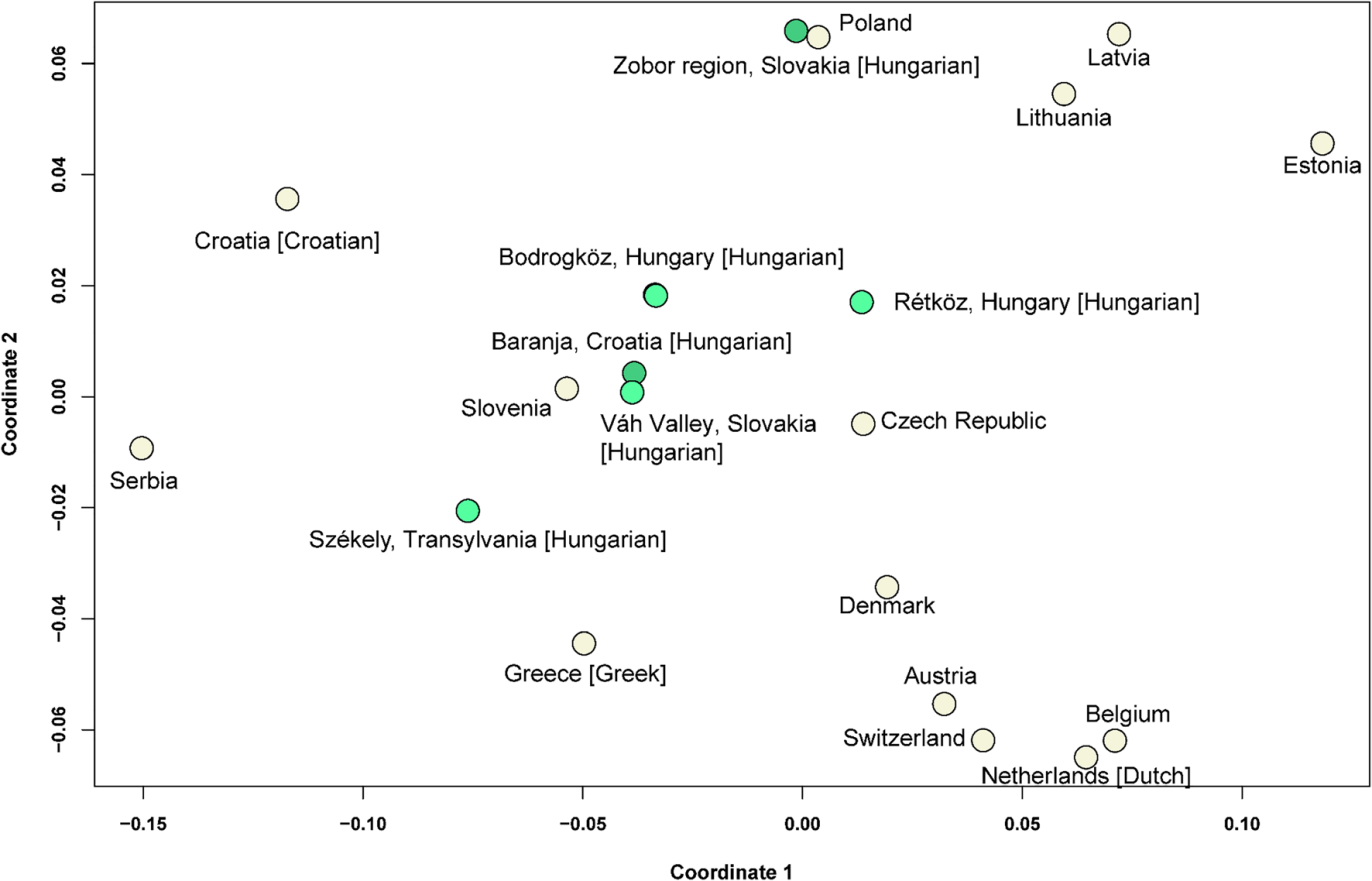
Non-metric MDS plot based on R_ST_ genetic distances. 23 loci Y-STR data of 7287 haplotypes from Baranja, Zobor region and neighboring population and other populations from Europe. Hungarian-speaking populations are indicated with green, dark green marks the here studied Baranja and Zobor region populations.

We further investigated these inter-population affinities with Y-STR data, calculating R_ST_ genetic distances. We constructed non-metric multidimensional scaling (MDS) plot based on Y-chromosomal haplotypes (*n* = 7287) collected from YHRD.org, consisting of 23 Y-STR loci from geographically relevant populations^40^ (Fig. 2). The R_ST_ genetic distances and R_ST_ *p* values of the studied populations are presented in Supplementary Table S9.

Whereas the Székely population still shows connections toward southern populations and to diverse groups of the Carpathian Basin (like nonsignificant distance from the Váh valley population and Baranja) and the Slovenians, the Baranja population shows a stronger genetic similarity to Bodrogköz, Váh valley, Slovenian and Czech populations beside the Székelys. Rétköz and Bodrogköz groups were the closest to Zobor region from the Carpathian Basin, although Polish population was also in nonsignificant distance (Supplementary Table S9).

Summarizing these results, we can conclude that the studied populations do not separate from their neighboring groups.  Although different trends are present in the two new datasets, a fine-scale geographic pattern and substantial genetic drift are not decipherable through grouped Y-haplogroup or 23 STR data analyses and low-resolution SNP typing.

### Phylogenetic analysis of the paternal lines

Based on the Y-STR haplotypes, median joining networks were constructed including samples from the two investigated regions (Fig. S4), and available 21 Y-STR datasets of the Carpathian Basin (Fig. S5). We can observe on these networks that the different paternal haplotypes are spread throughout the studied regions. Furthermore, based on the data reviewed to date, the Carpathian Basin does not display specific Y-haplotype structure in the modern male population that corresponds with its geography, aligning with the R_ST_ results. Only subtle differences are observable in the Székely population along with their shared paternal ancestries in the Bodrogköz/ Rétköz populations (through differences of frequencies of J2 and N1a haplotypes).

Subsequently, we analyzed specific haplogroups that can be linked to ancient Hungarian data from phylogenetic aspects.

We constructed eight networks (R1a-Z93, N-M46, R1b-P25/M343, G2a-L156, R1a-Z280, J2b-M12, C-M217 and I2a-P37) that are potentially helpful in uncovering the genetic legacy of the populations being studied. Six of them (N-M46, I2a-P37, G2a-L156, R1a-Z280, J2b-M12, C-M217) can be found with descriptions in Supplementary Information as Figs. S6–S12.

#### Median Joining network of 123 R1a-Z93 haplotypes

**Figure 3:**
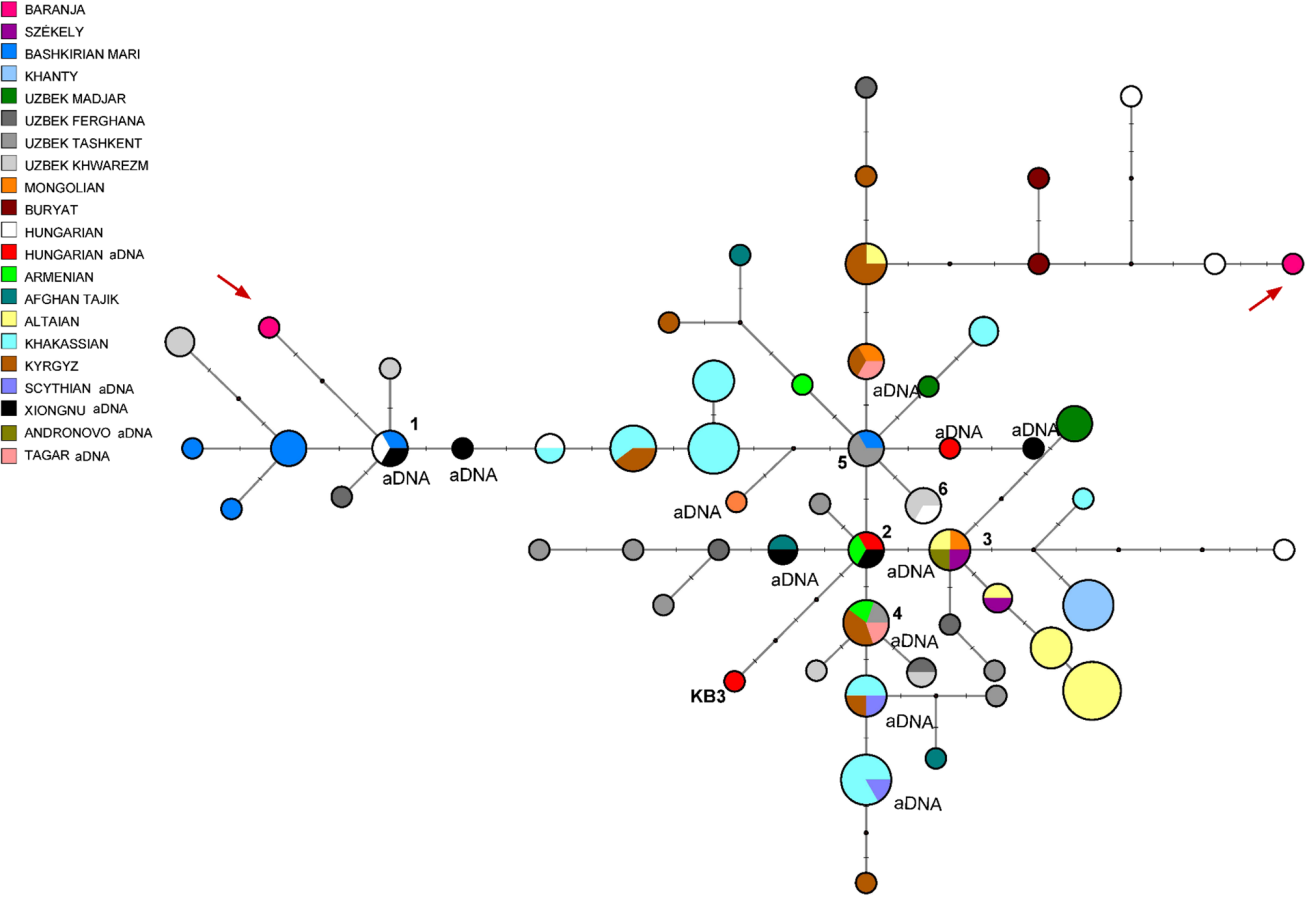
Median-Joining Network of 123 R1a-Z93 Y-STR haplotypes. The network was created with the Network (Fluxus-engineering) 10.2.0.0 program and the figures were drawn with the Network Publisher program. The circle sizes are proportional to the haplotype frequencies. The smallest area is equivalent to one individual. Arrows show the samples of this study. Cluster 2 includes three haplotypes: one Hungarian aDNA (Nagykőrös Gr2), one Xiongnu period aDNA (TUK09A) and one modern Armenian sample, separated by one molecular step (loci DYS389I and DYS390) from clusters 3 and 4. Cluster 2 is considered as the founding haplotype, as it contains two ancient haplotypes from males that lived 1000–2500 years ago. The Y-STR haplotype of Hungarian King Bela III (died in 1196 CE) is found three molecular steps away from cluster 2 (see KB3). Cluster 3 includes one Hungarian speaking Székely, one Mongolian, one Altaian, and one Bronze Age Andronovo aDNA (S10) samples. One ancient Hungarian sample (sample II54 from the Hungarian Royal Basilica of Székesfehérvár) is located one mutational step (DYS391) from cluster 5 and one mutational step (DYS19) from a Xiongnu period sample (TUK45).

A MJ network of 123 R1a-Z93 haplotypes from the 18 populations tested in this study or published previously from modern^23,30,41^ or ancient sources^18,42,43^ is seen on Fig. 3. Three modern Hungarian including one modern Baranja sample (DV 60) and one Xiongnu period (TUK04) aDNA samples, formed a common branch with Bashkirian Mari, Uzbek Khwarazm and Uzbek Fergana samples. One other Baranja haplotype is on a Central- and Inner-Asian branch, with two Hungarian samples.

The paragroup R1a-Z93* is most common in the Altai region of Southern Siberia nowadays, but it has also spread to Kyrgyzstan and all Iranian populations^41^. Furthermore, the R1a-Z93 haplogroup is also common in Tajik ethnic groups, in Afghan Pashtuns and Caucasus as well. Downstream haplogroup R1a-Z2125 occurres at highest frequencies in Kyrgyzstan and among Afghan Pashtuns^41^.

Keyser et al.^42^demonstrated that ancient Xiongnu period samples from Mongolia belonged to haplogroups R1a-Z93 (Z2125), which are also included in this network. One of them shared the same haplotype on 10 Y-STR level with a present-day Hungarian (see cluster 1 in Fig. 3) while another one with Hungarian aDNA (Nagykőrös Gr2) sample (see cluster 2 in Fig. 3), but they differ in deeper analyses when we consider their full available Y-STR profiles.

The Árpádian King Béla III, and another sample from Royal Basilica (II54) belonged to haplogroup R1a-Z93^18^, as well as two other Z93 samples were found among the Hungarian conqueror population as well^43^. On 15 Y-STR level King Béla III and a Xiongnu sample are three steps away from each other.

Although, Y-STR analyses of aDNA are challenging due to DNA degradation, SNP data are accumulating via whole genome sequencing and genome-wide capture approaches. We gained a more accurate haplogroup classification of the modern DV020 (Baranja) R1a-Z93 sample: it belongs to R1a1b2a2a3c2 ~ (FGC56440 terminal SNP), which subhaplogroup is found in the Hun period Carpathian Basin (Budapest Vezér street, Marosszentgyörgy in Romania), in middle-late Avar period samples and in Hun period Kazakhstan as well^44^. There are many examples of this haplogroup known from ancient genomic studies dated to the Bronze Age, found at Russian Krasnoyarsk, Kazakh Aktogay (1900–1400 BCE), and Early Iron Age Tasmola culture (700–500 BCE)^45^. Furthermore, some samples from the (Middle) Late Bronze Age Mongolia and a few Xiongnu samples also show the Z2124 subgroup based on whole genome SNP data^46,47^. The R1a1a1b2a2a3 ~ subhaplogroup is present in an early medieval Hungarian village cemetery site Homokmégy-Székes as well^7^. We conclude that this Y haplogroup might have arrived at the Carpathian Basin in one of the eastern migrations and can have an origin in the Kazakh steppe.

#### Median-joining network of 207 R1b-P25 Y-STR haplotypes

**Figure 4:**
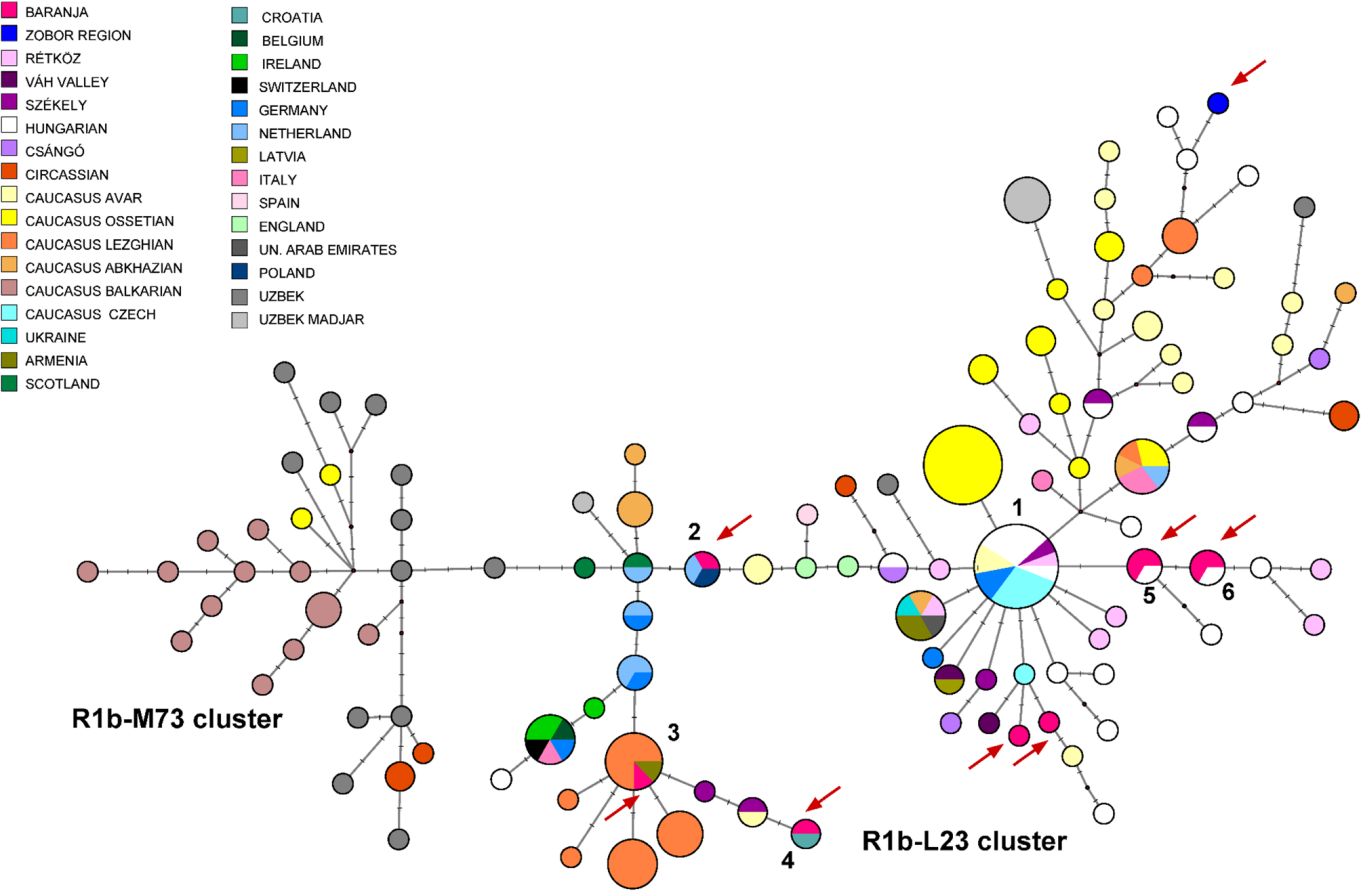
Median-Joining Network of 207 R1b*-P25 haplotypes. The circle sizes are proportional to the haplotype frequencies. The smallest circle is equivalent to one individual. Arrows show the samples of this study.

An R1b-P25 MJ network (with R1b-L23 and R1b-M73 subbranches) was constructed using 207 samples from the present study, from FTDNA data and populations previously studied^23,48,49^ (Fig. 4). All Hungarian haplotypes included in the network belong to the R1b-L23 cluster and most of them appear to be descended from the founding haplotype (cluster 1). In addition to the founding haplotype, some Hungarian haplotypes were shared with Europeans (cluster 2), or with Lezghians and Armenians from the Caucasus (cluster 3), as well as with Croatians (cluster 4). Hungarian, Belgian, Armenian, Croatian, German, and Scottish samples show the most similar haplotypes to Baranja and Zobor region samples within the R1b-L23 haplogroup, when compared at the 17 or 21 STR levels with samples from FTDNA.

Haplogroup R1b-M269 is the most frequent Western European lineage today. It was originally thought to have originated in the Paleolithic era, but recent studies suggest a Late Neolithic origin^6^. Most of the R1b-M412 chromosomes belong to Western Europeans, but another subgroup, R1b-L23 (xM412, R1b1a1b1), is commonly referred to as “Eastern European R1b”, prevalent in the Caucasus, Turkey, and Ural, with about 10% frequency^48^.

Olalde et al.^50^ have confirmed the role of R1b-L23 subclades in the expansion of the Eastern population of the Early Bronze Age Bell Beaker culture to Iberia, and it was also shown to be an important part of the Yamnaya culture-associated Early Bronze Age paternal ancestry^51^.

Based on recently published aDNA studies, haplogroup R1b-L23 was present in the territory of today’s Czechia and Poland in Corded Ware culture associated samples from 2000 to 3000 BCE^52^ , and later in the Hun period, in the Avars and the Hungarian conquerors^16,17,43,44^. Nowadays, examples of this subgroup are scattered throughout Europe, with the highest concentrations in the United Kingdom and Ireland, according to Yfull data.

In the two regions under investigation, five R1b-P25 samples were analyzed for the marker Z2103. All results fell under the Z2103 (R1b1a1b1b) subgroup. As this subgroup was both found in the Volga region during the eighth to fourteenth centuries (Szeifert et al. 2022) and in the local area in pre-Conquest times^15,53^, we cannot estimate its time of arrival in the Carpathian Basin. However, Baranja haplotypes from cluster 3 pinpoint a separate population event from the other clusters, most likely originating from the Caucasus.

Further detailed phylogenetic analyses of N-M46, I2a-P37, G2-L156, R1a-Z280, J2b-M12, and C-M217 median-joining networks can be found in Supplementary Information of this paper.

### Evaluation of the mitochondrial DNA data

#### Haplogroup-based analyses

Altogether 168 newly reported high-coverage whole mitogenomes were analyzed in this study, 79 from Zobor region and 89 from Baranja with a mean mitogenome coverage of 209.05×, using Illumina NGS technology.

The mitochondrial haplogroup frequencies of the two populations are presented in Table 2 and on Fig. S1.

**Table 2:** Major mtDNA haplogroups and their frequencies in the Zobor region and Baranja populations. Subhaplogroup resolution is detailed in Supplementary Table S3.

Haplogroup mtDNA	*n* (absolute frequency, Zobor)	Frequency Zobor region (%)	*n* (absolute frequency, Baranja)	Frequency Baranja region (%)
H	34	43.04	37	41.57
K	8	10.13	7	7.87
U5a	7	8.86	5	5.62
U2	5	6.33	2	2.25
J	4	5.06	7	7.87
T/ T1	4	5.06	3	3.37
U4	3	3.8	5	5.62
HV	2	2.53	3	3.37
T2	2	2.53	4	4.49
V	2	2.53	3	3.37
X	2	2.53	2	2.25
Y	2	2.53	0	0
D	1	1.27	0	0
N1	1	1.27	0	0
U5b	1	1.27	5	5.62
L	1	1.27	0	0
R	0	0	1	1.12
U3	0	0	2	2.25
W	0	0	3	3.37

In the Zobor region, 79 mitogenome sequences revealed 377 polymorphic sites, corresponding to 63 distinct haplotypes. These exhibited a haplotype diversity (Hd) of 0.9932. On the other hand, 89 mitogenome sequences of the Baranja region population displayed 447 variable sites, clustering into 78 unique haplotypes with a marginally elevated haplotype diversity of Hd = 0.9969 compared to the Zobor region.

**Figure 5:**
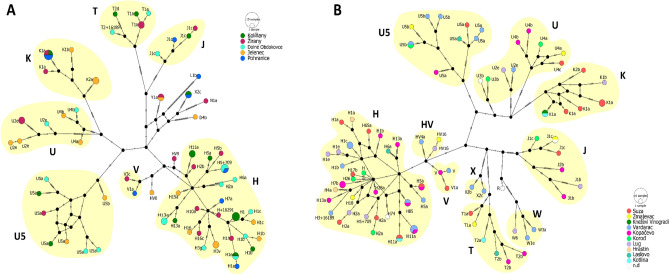
Median-joining network of modern-day mitogenomes (**A**) from the Zobor and (**B**) from the Baranja regions’ populations. The networks (nexus files) were created with DnaSP^54^ and the figures were drawn with the PopArt (Population Analysis with Reticulate Trees) program^55^.

The median-joining network of mitogenomes from the investigated regions showed a large variety of different haplogroups among the villages, without any unique pattern in either case (see Fig.  5). Most of the samples belong to the typically European H and U macrohaplogroups. Most of the haplogroups were shared among the villages, and almost all villages have diverse haplogroup distribution of the maternal lines in both studied regions. Notably, the U macrohaplogroup was absent in the samples from the Pohranice municipality in the Zobor region, which may be due to the limited sample size from this village. In the Baranja dataset, the majority of samples associated with haplogroup K originate from a single community, specifically Suza.

Due to the uneven and sometimes limited number of samples across villages, conducting an AMOVA test for among village heterogeneity wasn’t feasible. The variations however both within and between villages are distinctly illustrated in Fig. 5.

A single aDNA study from the ninth–twelfth century exists for the Zobor region, which served as a Hungarian-Slavic contact zone during that era. Although the ancient sample set is limited in size and restricted to hypervariable sequences, some parallels can be observed, notably within haplogroup U5a1b^56^. From the Baranja region, mostly prehistoric sample sets are published yet, which attest among others for the Neolithic presence of haplogroups T2a-b, K1a-b, and K2b in the area, and the prehistoric prevalence of J1c in the North Balkan^57^. These lineages are also found in today’s Baranja population.

Although most haplogroups in our samples align with those predominantly found in Europe, several outlier haplogroups were identified, including haplogroups L1b, N1a, X2, Y1a, D4, U4b, and U3b3. The appearance of outlier maternal lineage L1b in the Zobor data set is noteworthy. In Europe, mtDNA macrohaplogroup L represents less than 1% of the total population. L1b subgroup, dated at about 10 kya, has its frequency maximum in West Africa^5^. According to phylogeographic analyses carried out by Cerezo et al.^5^, around 65% of the European L lineages are believed to have arrived during more recent historical periods, such as the Roman period, the Arab conquest of the Iberian Peninsula and Sicily, and the Atlantic slave trade era. Ancient DNA data are scarce from these periods of Europe yet, therefore the origin of this group in the Zobor dataset remains open.

Although the mitochondrial N1a haplogroup was prevalent among the ancient Hungarians, the N1a representative from the Zobor region belongs to the prehistoric branch of the haplogroup (N1a1a1a3). The closest parallel to this lineage is from the southern area of Transdanubia (Western Hungary) and dates to the transition between the 6th and 5th millennia BCE (sample I0176 in^51^).

Haplogroup X2 occurs in two cases each from both regions (X2c1 and X2b). X2 is more prevalent in the populations of the Near East, Caucasus, and Mediterranean Europe, compared to those of northern and northeastern Europe, and rare among Eastern European populations. Furthermore, it is virtually absent in the Finno-Ugric and Turkic-speaking peoples residing in the Volga-Ural region^9,58^. Both detected subgroups have their parallels in prehistoric Europe, where X2b was more frequent. Two X2c1 samples from the Zobor region have close parallel from the Conquest Period Karos-Eperjesszög cemetery from northern Hungary (Karos 2/70)^14^.

The rare mitochondrial haplogroup Y1a is most probably a sign of the maternal continuity of the Avar population in the Zobor region, based on parallels in^15,44^. Besides the Avar period of the Carpathian Basin, aDNA haplogroup matches are only known from Mongolia and Kazakhstan^44,46,57^. Other outlier haplogroups (D4, U4b, U3b3) are discussed along phylogenetic analyses in the subsequent chapter.

**Figure 6:**
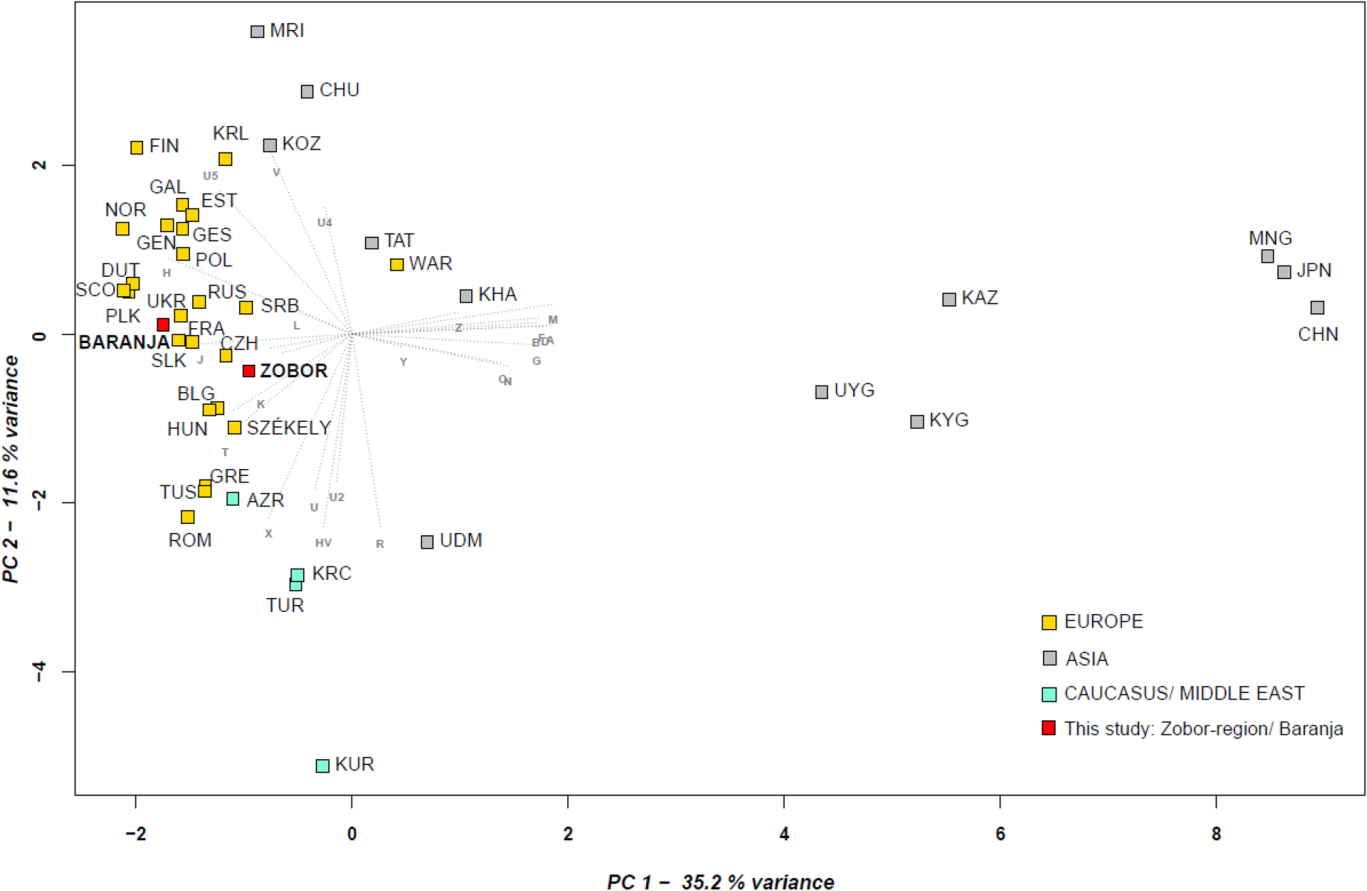
PCA plot with modern Eurasian populations. The PCA based on mitochondrial haplogroup frequencies captures the first and second principal components, presenting 46.8% of the total haplogroup variance. The investigated Zobor region and Baranja populations are indicated in red, the Europeans in yellow, Asians in gray and populations from the Caucasus and Middle East in light blue.

We used PCA to visualize the population genetic relatedness based on mtDNA profiles and haplogroup frequencies of 42 different populations (Supplementary Table S5 and Fig. 6).

The PCA positions both the Baranja and Zobor region datasets within the European cluster, aligning closely with the Czech and Slovakian populations. Subtle differences are observed between the Székelys, other published “average” Hungarians, and the groups of this study; notably, most of the East Eurasian haplogroups and haplogroup I are missing in the latter (Supplementary Table S5).

Due to the applied resolution of the haplogroup data, finer differentiation within this European cluster is not discernible.

#### Sequence-based analyses of the mitogenomes

We conducted a comprehensive examination of complete mitogenome, encompassing its 16,569 base pairs, through DNA sequence level analysis. Subsequently, Slatkin F_ST_ values were computed and documented in Supplementary Table S10. A heatmap, illustrating the clustering of F_ST_ values, was generated to elucidate the genetic differentiation among the populations under investigation (see Fig. S13). It is interesting that among the included Conquest Period aDNA datasets, the KL6 group–which stands for larger village cemeteries from the tenth to eleventh centuries^58^ –clusters with the Baranja, Hungarian, and Székely datasets.

The differences between the F_ST_ values are very small, whole mitochondrial data are missing from some neighboring regions and the Slovakian and Czech datasets are also limited; therefore, the resolution of that analysis is restricted to a broader scale. Mitogenome sequences from Hungarian populations from Hungary, Székely (Hungarian) people from Transylvania near Odorheiu Secuiesc, Romania^27^ and the here presented two populations from Baranja and Zobor region were tested in Arlequin for population differentiation and showed F_ST_ values below 0.0035 with significant *p* values.

We analyzed individual maternal lineages to discern the inter-regional relationships of contemporary Hungarian lineages and their ties to prehistoric and historic populations, among other associations. In the following we present those lineages that show diverse phylogenetic connections of the two study areas, including ancient reference samples as well (see references with non-NCBI IDs in Supplementary Tables S11).

On the NJ tree of haplogroup T1a, one individual from Baranja (DV082) can be found in the close proximity to one individual from archaeological site Bolshie Tigany (Volga-Kama region Early Medieval) on an excerpt of the phylogeographically very diverse and therefore less informative T1a tree (Fig. S14A). Another studied mitochondrial lineage from the Zobor region (ZB006) is situated close to an early medieval lineage from Bayanovo site in the Cis Ural region, associated with the late Lomovatovo culture (ninth–tenth centuries) and to one another sample from Bolshie Tigany (Fig. S14B). Whereas the structure of the whole T1a tree does not allow firm phylogenetic conclusions, these proximities on the tree might hint on the common history of these people (see further maternal connections of ancient Hungarians with the populations of the Cis Ural and Volga regions in Szeifert et al.^58^.

One sample from the Zobor region belongs to lineage D4b2b. While haplogroup D4 is predominantly found across East Asia, Southeast Asia, Siberia, Central Asia, and among the indigenous populations of the Americas, its presence in Europe is notably sparse^10^. The D4 mitogenome NJ tree (Fig. S15) shows that the D4b2b subgroup is rather disseminated in Eastern Eurasia nowadays. Although the currently known medieval ancient data (such as late medieval Mongolian sample) do not cluster with the examined ZB058, this lineage could reach the Carpathian Basin in the historically recorded migration weaves of the 1st millennium BCE.

The U2e phylogenetic tree highlights the diversity observed within the Zobor and Baranja regions (Fig. S16). While the Baranja sample DV023, classified under lineage U2e2a1a, demonstrates northern affiliations, two samples from the Zobor region do not neatly fit into any subgroups currently recognized in the phylotree (falling into the U2e1′2′3 category). Notably, samples from both the Zobor and Baranja regions share the U2e1b1a subgroup with individuals from the tenth to eleventh centuries in the Carpathian Basin. Furthermore, representatives from the Zobor region and the steppe, associated with the U2e1a1 subgroup, are also evident in the U2e tree (refer to Fig. S16).

The U3 phylogenetic tree indicates that the U3b and U3b3 lineages in Baranja have connections primarily to the south and east (Fig. S17), where ancient haplogroup matches also come from the Middle East and Caucasus^57^.

The U4 haplogroup evolved during the Last Glacial Period, and spread in Northern Eurasia, becoming a relatively common lineage among Mesolithic European hunter-gatherers^57^. On the U4 phylogenetic tree the Baranja samples have rather southern (Bulgarian, Serbian) connections whereas the Zobor region lineages show  connections toward Central and Eastern Europe (Fig. S18).

**Figure 7:**
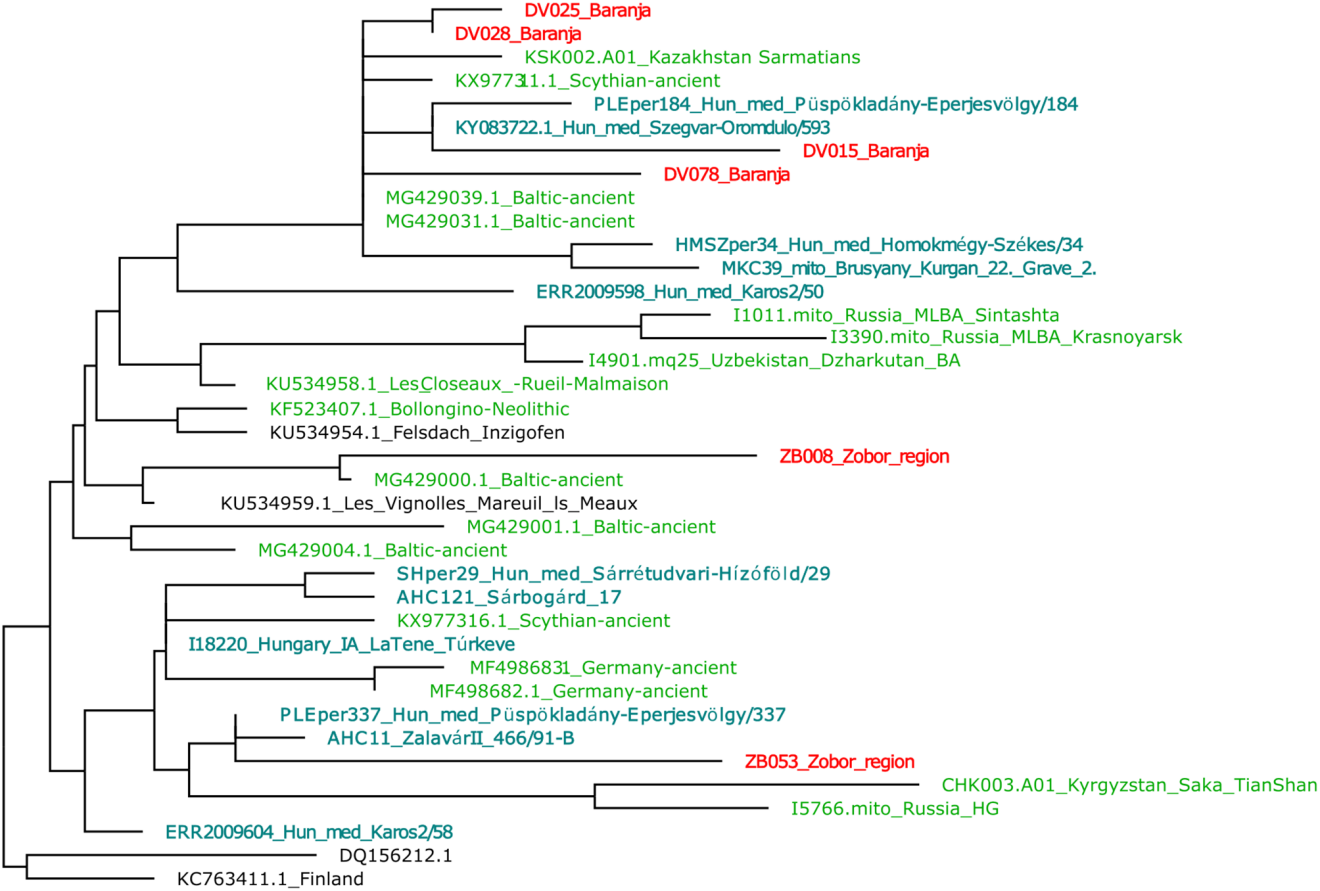
Partial neighbor-joining phylogenetic tree of mitochondrial subhaplogroup U5a2. The whole U5a tree is presented in Fig. S19. The red color indicates the samples from the investigated Baranja and Zobor regions and modern-day Hungarian-speakers, sample names with light green color are ancient samples, teal color indicates ancient samples from the territory of today’s Hungary or historically Hungarian-related samples.

The U5a haplogroup, prevalent across Western Eurasia, is also well-represented in the modern Carpathian Basin. Notably, its U5a2 subclade establishes a clear link with ancient samples from the closer and wider region, with important examples from the ninth to eleventh centuries cemeteries of ancient Hungarians (see Figs. 7 and S19).

The H13 haplogroup is present in both the Zobor region and Baranja, with pairs of individuals in each. However, their phylogeographic patterns differ strikingly (Fig. S20). In Baranja, the H13 lineages branch off basally, preceding most contemporary lineages. Conversely, in the Zobor region, lineages either match Northern European examples, as seen in the H13a1a1a lineage of ZB013, or are akin to a Roman-era sample from Dobrudja and modern Polish, Russian samples, as observed in the H13a1a3 lineages of ZB042 and ZB047.

## Discussion

In this study we aimed to elucidate the phylogenetic relationships of maternal and paternal lineages within rural Hungarian-speaking populations from Croatian Baranja and the Slovakian Zobor region. We further aimed to contrast these findings with those of other Eurasian populations and aDNA results, with a special focus on ancient Hungarians.

According to our findings both populations that their general uniparental compositions fit into the broader East-Central European context. Predominantly, the mitochondrial haplogroup distributions in both regions mirror those typically observed across Europe, with subtle differences among the regions. The phylogenetic structures of their lineages intersect and weave through each other, indicating a complex intertwined population history of the Hungarians. However, upon closer examination, subtle differences can be discerned for the populations of Baranja and the Zobor region. Both lack Q and H1a groups compared to other Hungarian datasets and have minimal amount of J. Whereas the Baranja region shows more connections to southern Europe, the Zobor region shares more lineages with northern and eastern neighbors. Notably, the Baranja population displays a conservation of paternal lineages also observed in the Hungarian conquerors, including haplogroups R1a-Z93, N-M46, R1b-L23, I2a-P37, and G2a-L156. Conversely, the genetic makeup of the Zobor region population exhibits subtly different characteristics in population genetic analyses and less diversity. Nevertheless, the reduced diversity observed could be attributed to the limited sample size, potentially skewing the Zobor region results. Furthermore, such relatively isolated populations are also more prone to genetic drift, which might lead to their differentiation. Previous studies show that Y-chromosomal haplotypes vary significantly across geographic regions, with more variation between population groups than within them compared to autosomal markers^49^. This pattern is attributed to the smaller effective population size of Y chromosomes, leading to stronger genetic drift and haplotype clustering due to widespread patrilocality. As a result, Y-chromosomal genetic databases demonstrate pronounced population structure. The NRY, influenced by its small effective population size and patrilineal cultural practices, exhibits the highest genetic differentiation over geographic distances among genomic regions.

The phylogenetic analyses showed that Hungarian-speaking males share certain common haplotypes with ancient Xiongnu, ancient Avar, and Caucasian males within haplogroups R1a-Z93, G2-L156, and R1b-L23, suggesting a minor common genetic footprint. On the other hand, subgroups like R1a-Z280 and N1a1-Tat connects Hungarians and the here presented populations to the Ural region. Interestingly, the Y-STR pattern of the I2a prevalent in the South-Eastern European context, and also the whole Y-STR networks show predominant admixture of the Hungarian populations during their common history. The genetic homogenization happened not only within the Hungarians, but also with the surrounding populations as shown in this study.

The comprehensive evaluation of 168 high-coverage whole mitogenomes from the Zobor and Baranja regions provides invaluable insight into the maternal genetic landscape of the Carpathian Basin, previously detailed to this extent only in study of the  Székelys. Mitogenome-level comparisons stress the shared heritage and interconnectedness of the previously studied bulk of Hungarian populations, now including those from the Baranja and Zobor regions.

However, the presence of certain outlier maternal haplogroups (like L1b, N1a, X2, Y1a, D4, U4b, and U3b3) in Baranja and Zobor region attests to more complex historical and prehistoric interactions. The neighbor-joining phylogenetic analyses of selected lineages provide a clearer understanding of their genetic affiliations with historic and prehistoric samples and other modern-day groups. The genetic patterns seen, such as on the U4 or H13 lineages, suggest that those detected in Baranja are related to southern regions or have ancient local origins, while the Zobor lineages appear to be connected to far-off areas such as Central, North and Eastern territories of Europe. The phylogenetic analyses conducted on specific maternal lineages, particularly U2e1 and U5a1, have revealed noteworthy connections with genetic samples associated with the tenth–eleventh centuries Hungarians. Nevertheless, lineages of Eastern Eurasian origin (possible Avar legacy) and traces to the early Hungarians were also found in a small number in the Zobor region population (e.g. D4 and T1a haplogroups).

Comparing the Y-chromosomal diversity with the mitochondrial one in a comprehensive way, we experienced high haplotype diversity values,exceeding 0.99, in both the maternal and paternal gene pools. However, the diversity in male lineage was slightly lower in the Zobor region. We can conclude that local pre-Conquest Period and ancient Hungarian-related lineages occurred in both gene pools. The Eastern Eurasian lineages are more hidden in the Y-chromosomal system than in the maternal one, and therefore their localization needs more care. Subgroup level analyses of Y-chromosomal R1b-P25, for example, reveal diverse phylogenetic origins within the studied communities. In summary, more haplogroups of Eastern Eurasian origin (C-M216, N-VL29, R1a-M198*, R1a-Z93) in paternal types could be detected among the two groups than in maternal lineages (U4a2, Y1a1, D4b2b), with total frequencies of 4.9% and 3.5%, respectively. However, both pools lack notable Eastern types described in the Székelys previously (paternal Q, J and maternal A, B, C). Haplotypes with Near Eastern or Caucasian origin were found in both the maternal and paternal pools in a greater number than East Eurasian haplogroups (16.4% and 27.5%).

We have to note that modern day genetic data allows only limited possibility to derive historical conclusions, especially on sex-specific population events. Ancient genetic data from here discussed time horizons demonstrated that both Asian Avars and Hungarian conquerors arrived in families^59,60^. How these balances altered during the medieval and modern era is a study for future research.

We note furthermore, that despite thorough documentation of the sample donors and their ancestors, sampling biases may still occur. It would be advantageous to examine samples from as many predominantly Hungarian-speaking small settlements as possible. However, given the constraints—while avoiding sampling known relatives and excluding recent migrations—we aimed to compile a dataset that best represents the region.

Neighbor Joining and Median Joining methods are commonly used to construct phylogenetic trees based on genetic distance matrices. Considering that selected samples are taken from specific populations, the phylogenetic trees do not represent processes affecting the entire population and may not capture complex population histories, such as admixture events or population bottlenecks. Nonetheless, they can still provide useful clues tracing individual lineages.

In summation, this paper provides a detailed analysis of the paternal and maternal genetic heritage of the Hungarians in the Zobor and Baranja regions. These areas were selected based on the well-documented historical presence of Hungarians and their relative cultural isolation over the last centuries. By selecting historically recorded communities from several villages, the study sample is considered representative of these two regions. Combined with our previous study on the Székelys, it contributes to a broader understanding of the 19th-century population of the Carpathian Basin. Diverse lineages found among these communities echoing millennia of migrations, interactions, and genetic amalgamations. This study set an example for detailed regional and lineage-based studies within specific geographical regions.

Further investigations are imperative to determine how shared genetic markers were aquired by different groups. For a more comprehensive understanding of the origins, expansion, and ethno-linguistic associations of these populations, it is essential to undertake in-depth studies that include other European, Central Asian, and Caucasian populations. These studies should emphasize deep phylogeny, employing downstream SNPs and NGS techniques on the Y-chromosomes, and feature dense sampling across various regions and cultural groups. Comparing these enriched medieval datasets with future datasets researchers can test the dynamics of neighboring and subsequent populations and examine the transformations brought about by population influx events. Similar meticulously curated dense uniparental databases from the Carpathian Basin not only enrich our understanding of the population history of the region but also provide a pivotal reference point for European demographic studies of the 2nd millennium CE.

## Methods

### Testing of Y-STR and Y-SNP markers

The DNA was extracted from buccal swabs using the QIAamp DNA Mini Kit (Qiagen) following the spin protocol recommended by the manufacturer. The concentration of the extracted DNA for mtDNA workflow was measured using the QubitTM dsDNA High Sensitivity Assay Kit (Thermo Fisher Scientific, Waltham, MA, USA).

The samples were quantified for Y DNA workflow using the Quantifiler Human kit and the ABI 7500 Real-time PCR System (Thermo Fisher Scientific, Waltham, MA, USA).

DNA from the Baranja and Zobor region populations was surveyed for genetic variation using the Promega PowerPlex Y23 kit. Allele sizing and calling were determined with the ABI3500 Genetic Analyzer and GeneMapper ID-X v.1.4 software. To test for Y-SNP markers, we performed amplifications of 1–2 ng genomic DNA with Custom TaqMan probes and analyzed the relative fluorescence of the PCR products in an ABI 7500 Real-time PCR instrument using SDS.1.2.3/HID software. The SNP markers tested were CT-M168, KT-M9, PR-M45, T-M170, I-M170, I1-M253, I2b-M223, I2a-P37, J-M304, J1-M267, J2-M172, J2-M67, J2b-M12, R-M207, R1-M173, R2-M124, R1a-M198, R1a-SRY1083.1, R1a-M458, R1a-Z93, R1a-Z280, R1b-M343, R1b-P25, R1b-U106, R1b-P312, R1b-M412, R1b-Z2103, D-M174, N-M231, N1-LLy22g, N-L708, N-M46, N-L1034, N-VL29, N-Z1936, N-F4205, R1a-Y2633, and N-Y24365. The haplogroups are described in accordance with the generally accepted nomenclature, as it is common practice^3,61^. A complete list of primers and Taqman probes for binary markers is included in Supplementary Table S2. Haplotype and haplogroup frequencies and their diversity values were calculated using the formula from Nei (1973)^62,63^.

The Y-STR haplotypes in this study were submitted to the Y Chromosome Haplotype Reference Database, YHRD (yhrd.org, accession numbers: YA006013 for the Baranja and YA006014 for the Zobor region populations)^64^.

To provide a more accurate classification for some of the samples from R1a-Z93, R1b-P25*, R1a-M198*, I2a-P37, M343* subgroups, we used SNP tests from YSEQ GmBH^65^. Most specific positions on the YFull YTree v11.04^66^ in these cases are included in Supplementary Table S3.

### Phylogenetic analysis of the Y-STR haplotypes

To examine the STR variation within the haplogroups, Median Joining (MJ) networks were constructed using the Network 10.2.0.0 program and the figures were drawn with the Network Publisher 2.1.2.5 program^67,68^. Repeats of the DYS389I locus were subtracted from the DYS389II locus, and the DYS385 locus was excluded in every case because the Network program cannot handle the duplicated loci. To put the results into a more extensive geographical context, we included haplotypes of ten overlapping evolutionarily stable STR loci (DYS389I, DYS389II, DYS390, DYS391, DYS392, DYS393, DYS19, DYS437, DYS438, DYS439) from other Eurasian populations. In some cases, where more high-resolution data were available, we also created networks using 15, 17 or 21 STRs.

### Paternal genetic structures

Based on the 23 Y-STR haplotypes, pairwise R_ST_genetic distances were computed with YHRD.org’s online AMOVA, and the MDS plot was constructed in R^64^. We used both R_ST_ and F_ST_-based MDS analyses for population comparisons, since F_ST_ is more efficient when there are high levels of gene flow, whereas R_ST_ (an analogy of F_ST_ based on allele size difference) reflects population differentiation better under low gene flow^69^.

Pairwise F_ST_genetic distances were calculated based on haplogroup frequencies using Arlequin 3.5 software^70^. Slatkin’s linearized F_ST_ distances were computed in Arlequin from haplogroup distributions as allele frequency data. We used these linearized Slatkin F_ST_ values for clustering in Python using the seaborn *clustermap* function (parameters: metric = ‘*correlation*’, method = ‘*complete*’)^71^.

### Methods of mtDNA analyses

#### DNA extraction, library preparation and sequencing

The amplification of the entire mitochondrial DNA (mtDNA) was carried out using the ExpandTM Long Range dNTPack kit (Sigma Aldrich), following the protocol described by Fendt et al.^72^. The primers used for amplification were as follows: forward ‘A’ (FA) 5′–3′: AAATCTTACCCCGCCTGTTT, reverse ‘A’ (RA): AATTAGGCTGTGGGTGGTTG, forward ‘B’ (FB): GCCATACTAGTCTTTGCCGC, and reverse ‘B’ (RB): GGCAGGTCAATTTCACTGGT. The mtDNA was amplified in two fragments, and the PCR program was adjusted based on the fragment length. The long-range PCR conditions included an initial denaturation step at 92 °C for 2 min, followed by 10 cycles of denaturation at 92 °C for 10 s, annealing at 60 °C for 15 s, and elongation at 68 °C for 8 min 30 s, 10 cycles of denaturation at 92 °C for 10 s, annealing at 60 °C for 15 s, and elongation at 68 °C for 8 min 50 s, 15 cycles of denaturation at 92 °C for 10 s, annealing at 60 °C for 15 s, and elongation at 68 °C for 9 min 10 s, and a final elongation step at 68 °C for 7 min. The PCR products were checked on an agarose gel stained with EcoSafe and visualized using UV transillumination. The separately amplified fragments were pooled and purified using the QIAquick PCR Purification Kit (Qiagen), and the concentration of the purified PCR products was measured using the QubitTM dsDNA Broad Range Assay Kit (Thermo Fisher Scientific).

For library preparation, the NEBNext Ultra II FS DNA Library Prep Kit was used to prepare the mtDNA libraries. The quality of the library products was assessed using the Agilent D1000 ScreenTape Assay on the 4200 Tapestation system. Next-generation sequencing (NGS) was performed on the Illumina Miseq System (Illumina) using the Illumina Miseq Reagent Kit V2 (2 × 150 cycles) sequencing kit. The final concentrations of the indexed libraries were adjusted to 4 nM, considering the desired coverage to be achieved. To increase sample heterogeneity, 5% PhiX was added to the pooled samples.

#### Pre-processing of the sequencing data

The Illumina sequencing data was processed using a custom in-house bioinformatic pipeline^73^. Paired-end reads were merged using SeqPrep master^74^ with a maximum of one mismatch allowed. The base with higher quality was chosen in case of mismatches and reads with two or more mismatches were discarded. The pre-processed reads were then mapped to the rCRS reference sequence (NCBI Reference Sequence: NC_012920.1) using BWA v.0.7.5^75^ with a MAPQ threshold of 30. Consensus sequences were called using the majority rule for high coverage mitogenomes, without examining indels in the process. Samtools v.1.3.1^76^ was used for additional data processing tasks such as indexing, removal of PCR duplications, and creation of bcf files.

Mitochondrial haplogroup determinations were performed using HaploGrep2^77^, which utilizes Phylotree mtDNA tree Build 17^78^.

#### Heteroplasmy and NUMT detection

We employed the Mutserve program for heteroplasmy detection, as per^79^ Weissensteiner, Forer, et al. (2016) which can identify heteroplasmy down to 1% (Supplementary Table S4). Albayrak et al.^80^ suggested that mtDNA heteroplasmies under 2% might be NUMT (nuclear mitochondrial DNA) segments and should be separated from genuine heteroplasmy. The presence of NUMT amplicons of the same size could potentially signal heteroplasmy levels in the amplicon mix. To cleanse the large target mtDNA amplicons from potential NUMTs short PCR products, we utilized long-range PCR before sequencing, as described by Sobenin et al.^81^. Since we amplified mtDNA using long-range PCR, we only cross-referenced the longer (longer than 1700 base pairs) NUMTs from Dayama et al.^82^ using BLAST. This was to ascertain if any of our primers for long-range PCR might interact with them. Our findings revealed no areas of direct binding.

#### Phylogenetic analysis of the mtDNA

To construct neighbor-joining mtDNA phylogenetic trees, we sourced all mtDNA sequences accessible from public databases, with the majority coming from the NCBI database. From this collection, we retained only those sequences that shared a similar or identical haplotype to our samples. This refined dataset was then categorized into broader clusters based on their haplogroups. We aligned sequences in each group with ClustalO within SeaView^83^. The alignments were checked and corrected manually where necessary. Comparing to the rCRS sequence, we deleted the following ambiguous base positions 42, 57, 291–317, 447–458, 511–524, 568–573, 594–597, 1718, 2217–2226, 3106–3110, 3159–3167, 5890–5894, 8272–8281, 16,184–16,193. Next, neighbor-joining (NJ) trees were generated by PHYLIP version 3.6.^84^. The phylogenetic trees were drawn using Figtree version 1.4.2.^85^.

#### Population genetic analysis of the mtDNA

Principal component analysis (PCA) was performed based on the mtDNA haplogroup frequencies of 43 modern populations (see the list of populations and haplogroup frequencies in Supplementary Table S5). In the PCA of the modern populations, we considered 25 mitochondrial haplogroups, which are detected in the study population and in the relevant comparative populations and provide a broad-scale description of the analyzed maternal diversity within the present-day Eurasian context. The PCAs were carried out using the prcomp function in R v4.0.0.^86^ and visualized in two-dimensional plots with two principal components (PC1 and PC2).

We calculated population pairwise F_ST_ and linearized Slatkin F_ST_ values based on the whole mitochondrial genome sequences of genetically and geographically relevant modern-day individuals (classified into 28 groups) and ancient individuals (classified into 3 Hungarian conqueror groups, KL4-5-6 which indicate different cemetery types in the Hungarian Conquest Period, as used in Szeifert et al. (2022)) using Arlequin v3.5.2.2.^70^ with the following settings: Tamura & Nei substitution model with 10,000 permutations, a significance level of 0.05, and a γ value of 0.3.

We used the same linearized Slatkin F_ST_ values for clustering in Python using the seaborn clustermap function (parameters: metric = ‘*correlation*’, method = ‘*complete*’)^71^.

### Ethics approval and consent to participate

All procedures performed in the studies involving human participants were in accordance with the ethical standards of the 1964 Helsinki Declaration and its later amendments. For sampling, handling and storage of personal data and genetic samples, we adhered to the Hungarian 2008/XXI. law as guidelines. The Hungarian 2011/CXII. law provided us rules about the information and self-determination rights of the sample providers. The Data Protection Code on Data Protection Standards for Research Activities of the Research Centre for the Humanities (MTA BTK-KP/450-17/2018) was taken into account during the research. Before our sample collection, we asked the Hungarian Deputy State Secretary for National Medical Officers in the Ministry of Human Capacities, and the Committee on Research Ethics for authorisation. The Hungarian Medical Research Ethics Committee has confirmed that no ethical approval is required, because our study has no clinical relevance (our sample donors are not patients, data were not collected for health studies or medical research). None of the participant institutes had a Research Ethical Committee at the time of the sampling. We believe that our study does not contain case studies using individual people with identifying information/ personal data with the unique identifiers. They cannot be re-identified on the basis of the article. Sampling was entirely voluntary, signed informed consent forms to participate and to publish from each of the sample donors are provided.

### Supplementary Information

Supplementary Figures.

Supplementary Tables.

Supplementary Information.

## Data Availability

The data presented in this study are openly available in the EMBL Nucleotide Sequence Database (ENA) at [https://www.ebi.ac.uk/ena/browser/search], accession number [PRJEB64294] regarding the mitogenome sequences and at Y-STR Haplotype Reference Database (YHRD), accession numbers: for samples from the Baranja region: YA006013 [https://yhrd.org/YA006013]; for samples from the Zobor region: YA006014 [https://yhrd.org/YA006014]. All other underlying data are presented in Supplementary Tables of this paper.
