# Supplementary material for: Phylogenetic insights into the genetic legacies of Hungarian-speaking communities in the Carpathian Basin
Source: Sci Rep. 2024 May 20;14:11480. doi: 10.1038/s41598-024-61978-4 (PMC11106325; doi:10.1038/s41598-024-61978-4)

Fig. S16 Neighbor-joining phylogenetic tree of mitochondrial haplogroup U2e

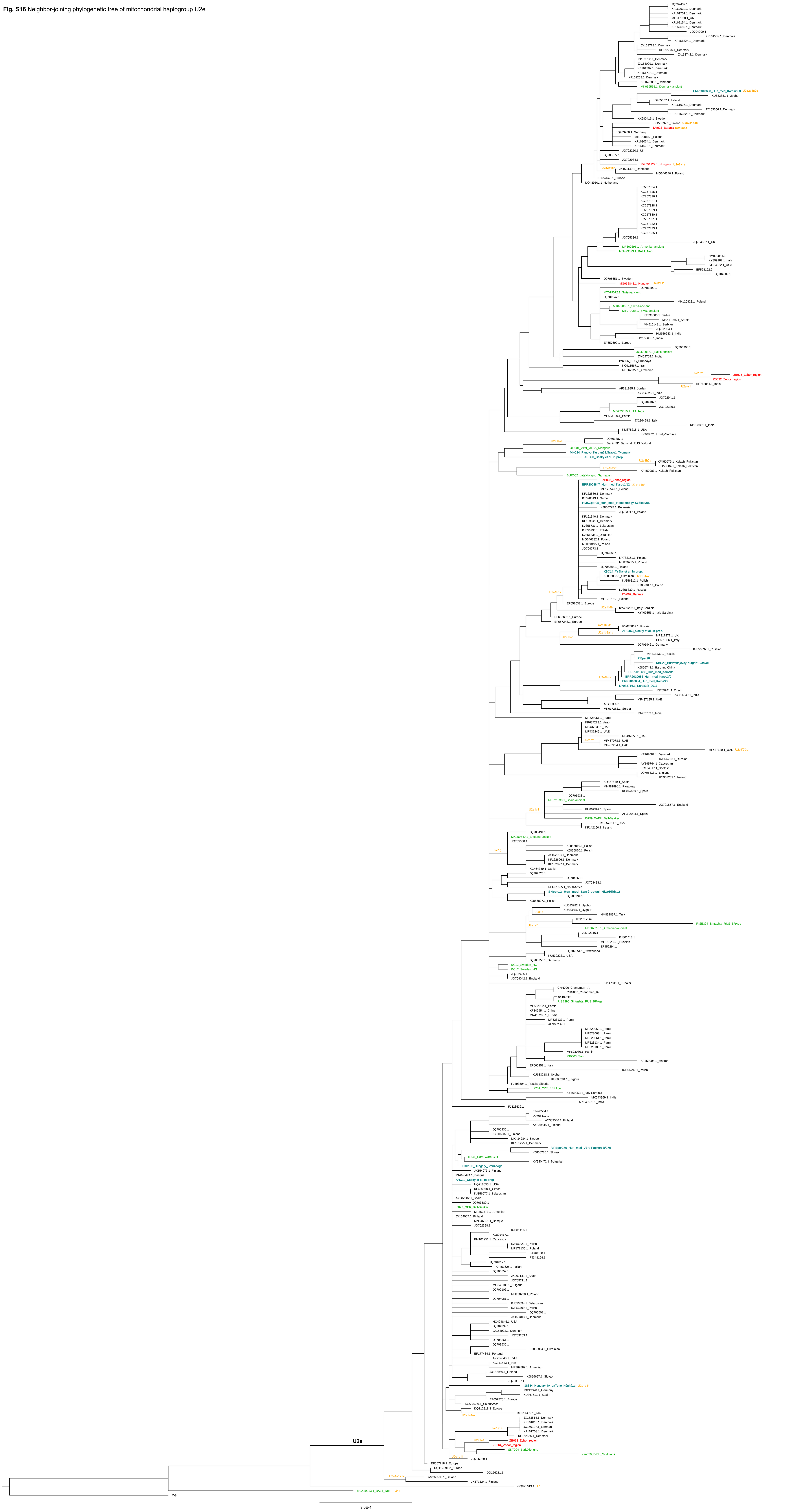

Fig. S17 Neighbor-joining phylogenetic tree of mitochondrial haplogroup U3

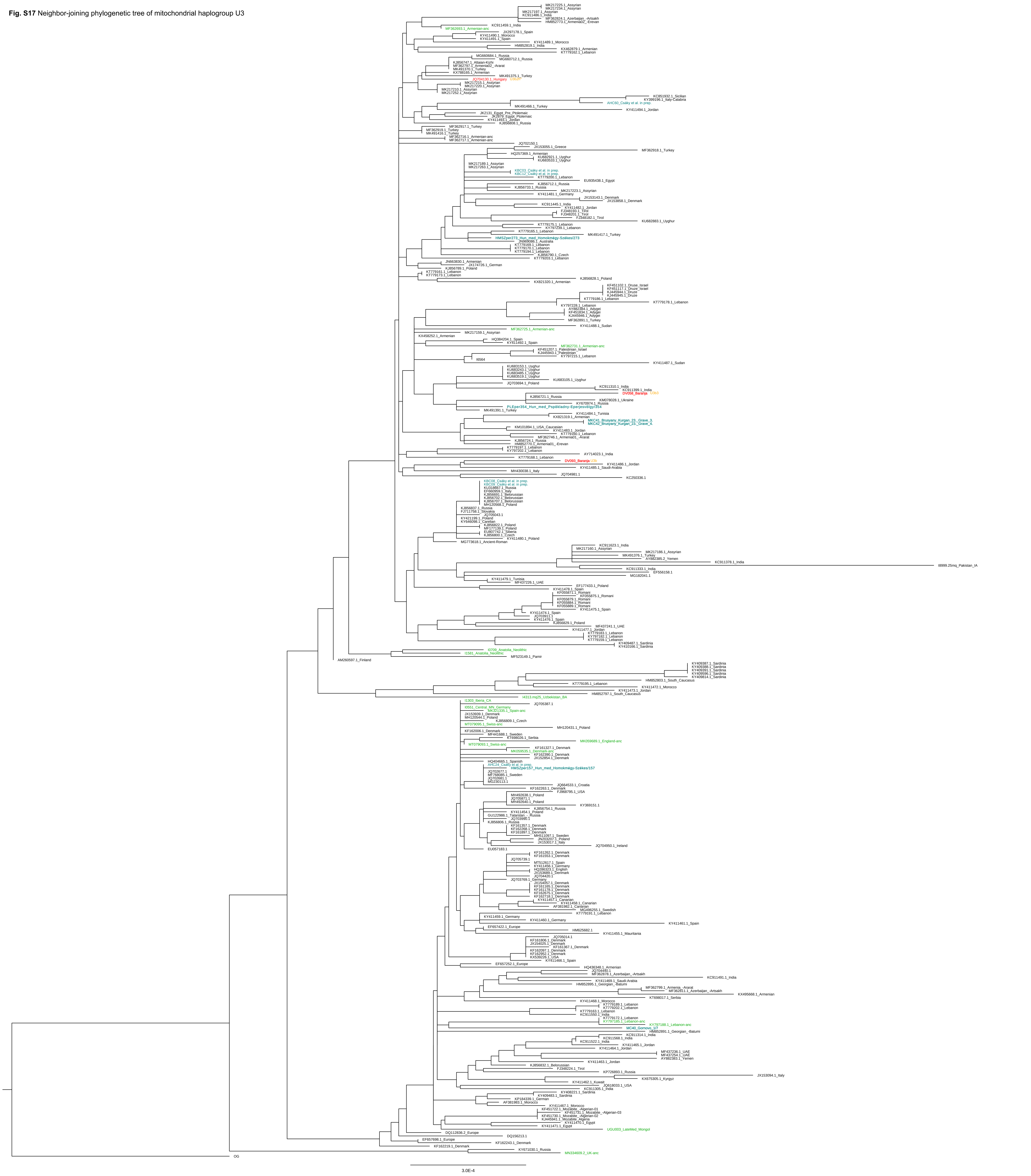

Fig. S18 Neighbor-joining phylogenetic tree of mitochondrial haplogroup U4

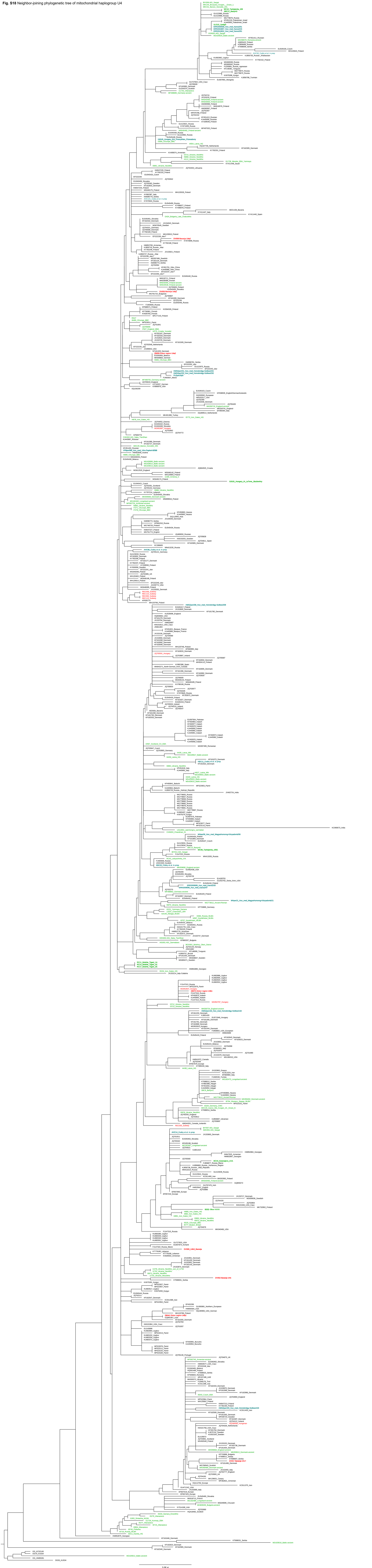

Fig. S19 Neighbor-joining phylogenetic tree of mitochondrial haplogroup U5a

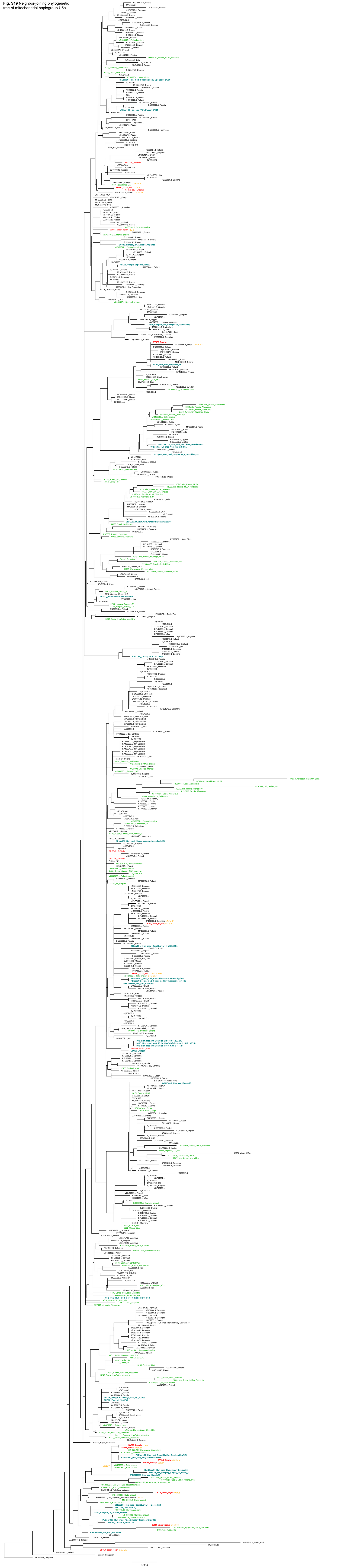

Fig. S20 Neighbor-joining phylogenetic tree of mitochondrial haplogroup H13

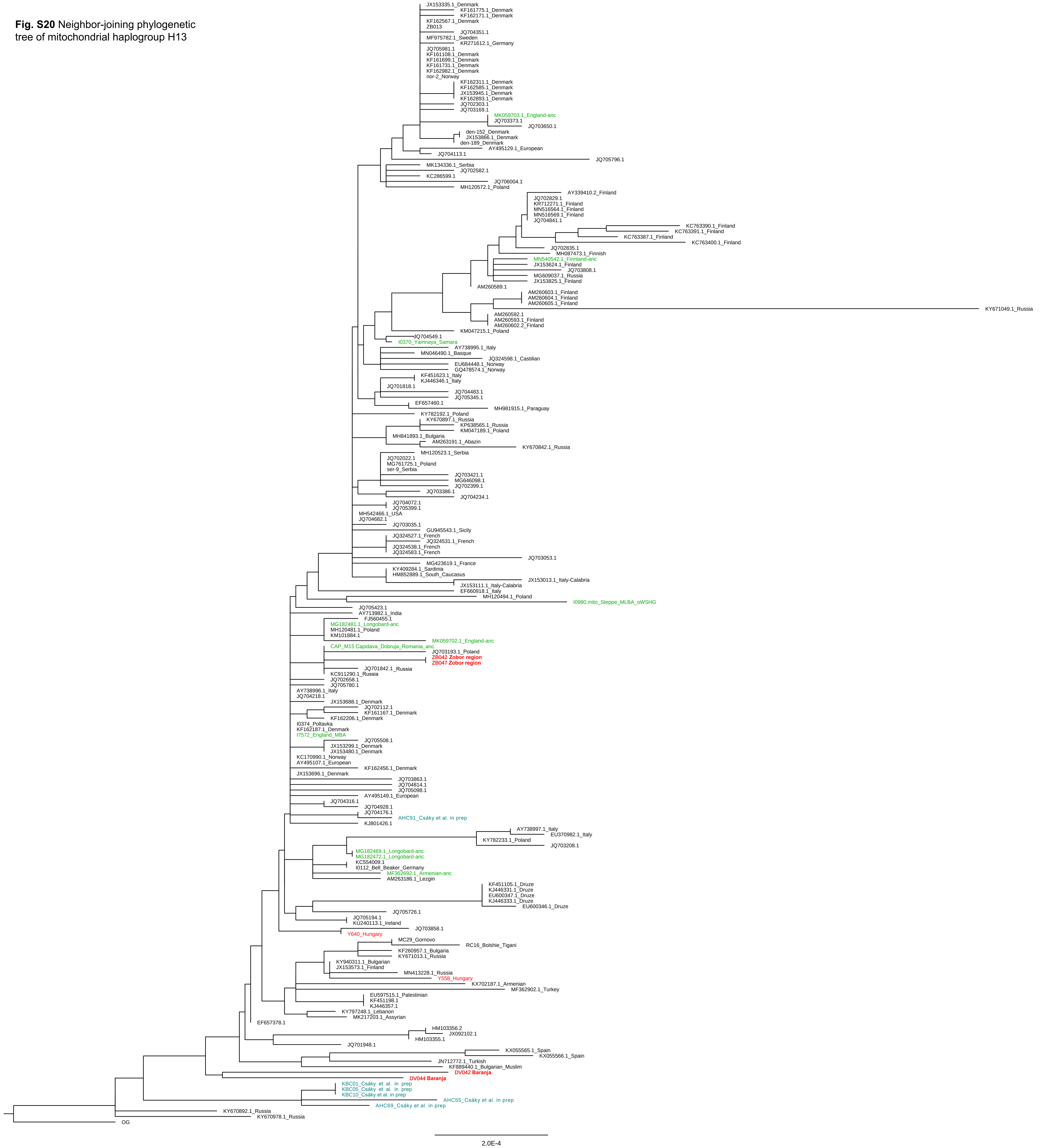

Supplement: Supplementary file 1 — Supplementary Figures. [file 41598_2024_61978_MOESM1_ESM.pdf]
