# Supplementary material for: Phylogenetic insights into the genetic legacies of Hungarian-speaking communities in the Carpathian Basin
Source: Sci Rep. 2024 May 20;14:11480. doi: 10.1038/s41598-024-61978-4 (PMC11106325; doi:10.1038/s41598-024-61978-4)
Supplement: Supplementary file 3 — Supplementary Information. [file 41598_2024_61978_MOESM3_ESM.docx]

**Phylogenetic Insights into the Genetic Legacies of Hungarian-Speaking Communities in the Carpathian Basin**

Noémi Borbély^1,2^, Dániel Dudás^3^, Attila Tapasztó^3^, Eszter Dudás-Boda^3^, Veronika Csáky^1^, Bea Szeifert^1^, Balázs Gusztáv Mende^1^, Balázs Egyed^4^, Anna Szécsényi-Nagy^1^* & Horolma Pamjav^3^*

^1^ Institute of Archaeogenomics, HUN-REN Research Centre for the Humanities, Budapest, 1097 Tóth Kálmán utca 4, Hungary

^2^ Doctoral School of Biology, Institute of Biology, ELTE Eötvös Loránd University, Budapest, 1117 Budapest Pázmány Péter sétány 1/C, Hungary

^3^ Department of Reference Sample Analysis, Institute of Forensic Genetics, Hungarian Institute for Forensic Sciences, Budapest, 1027 Gyorskocsi u. 25, Hungary

^4^ Department of Genetics, ELTE Eötvös Loránd University, 1117 Budapest Pázmány Péter sétány 1/C, Hungary

*Correspondence: Anna Szécsényi-Nagy and Horolma Pamjav

E-mail addresses of corresponding authors: [szecsenyi-nagy.anna@abtk.hu](mailto:szecsenyi-nagy.anna@abtk.hu); phorolma@hotmail.com

**Supplementary Information and Figures**

**Section 1**

**Ethnohistorical description of the investigated regions**

The Baranja (Drávaszög) region is geographically small, yet its historical past, folk traditions, and present human landscape are rich, diverse, and valuable. Depopulation characterizes the area, with approximately 5,000 Hungarian inhabitants residing there. Its history dates back to the 9th-10th centuries, associated with the Hungarians; the Hungarian Conquerors settled along the Danube, making it less populated compared to other regions. Reliable data became available from the time of written records and charters. 13th-century border descriptions already mention settlements as Hungarian: Bilje (Bellye), Suza (Csúza), Vardarac (Várdaróc), Kneževi Vinogradi (Hercegszőlős), Kopačevo (Kopács), Kamenac (Kő), Lug (Laskó), Kotlina (Sepse), Zmajevac (Vörösmart). They remained Hungarian during the Turkish era. Isolated from roads and wars, communities managed to preserve their Hungarian identity, including those listed. These settlements are predominantly Hungarian; during our sample collection, we covered most of these. They preserved folk characteristics that have disappeared or been assimilated in other Hungarian communities. Ethnographic peculiarities, such as fishing and wood processing in Kopácsi, were novelties for ethnographic research until recently^1^. Rural community life here retains traditions, including rich wedding and carnival customs, and parades. Some customs resemble those in the Zobor region near Nitra. Beliefs and folk tales have similarities to those found only in Székely Land, Transylvania. There is an abundance of riddles (4,800), proverbs (12,000), children's rhymes, and toys^2^.

The assimilation of Hungarians is very high; the Hungarian population decreased by 30% in 10 years. In mixed marriages, maintaining Croatian/Serbian ethnicity is a common practice, often resulting in the loss of the Hungarian language by the third generation^1^. This knowledge strengthens our well-documented collection; those who identified as Hungarian and had only Hungarian ancestors representatively embody the community we aim to sample.

The Hungarian population of the Nitra (Zobor) region is indigenous and dates back to the Árpádian period. The settlement of the area by Hungarians was a lengthy process. By the 11th and 12th centuries, Hungarian settlements were already interspersed among the Slavic settlements in the foothills, and by the mid-13th century, the foundations of the present settlement pattern were established, leading to the gradual loss of the Slavic majority in the Zobor region^3^. The Hungarian expansion after the conquest reached its peak in the 13th century, constituting the maximum extent of Hungarian ethnic territory in this area. Following the Mongol invasion, Slavic resettlement and expansion strengthened, although the regional structure established by the end of the 13th century remained largely unchanged until the mid-17th century^4^.

During the Turkish (Ottoman) wars, the ethnic composition of the Nitra region underwent significant changes. The predominantly Hungarian villages located in the river valleys and plains suffered significantly more during the prolonged conflicts than the Slovakian villages situated in the more sheltered mountainous areas^5^. Over the following 100 years, the increase in the Slovak population in the Nitra district was directly proportional to the decrease in the Hungarian population^6^.

**Haplogroup frequency distributions in the investigated populations and other Hungarian groups**

**A**

**
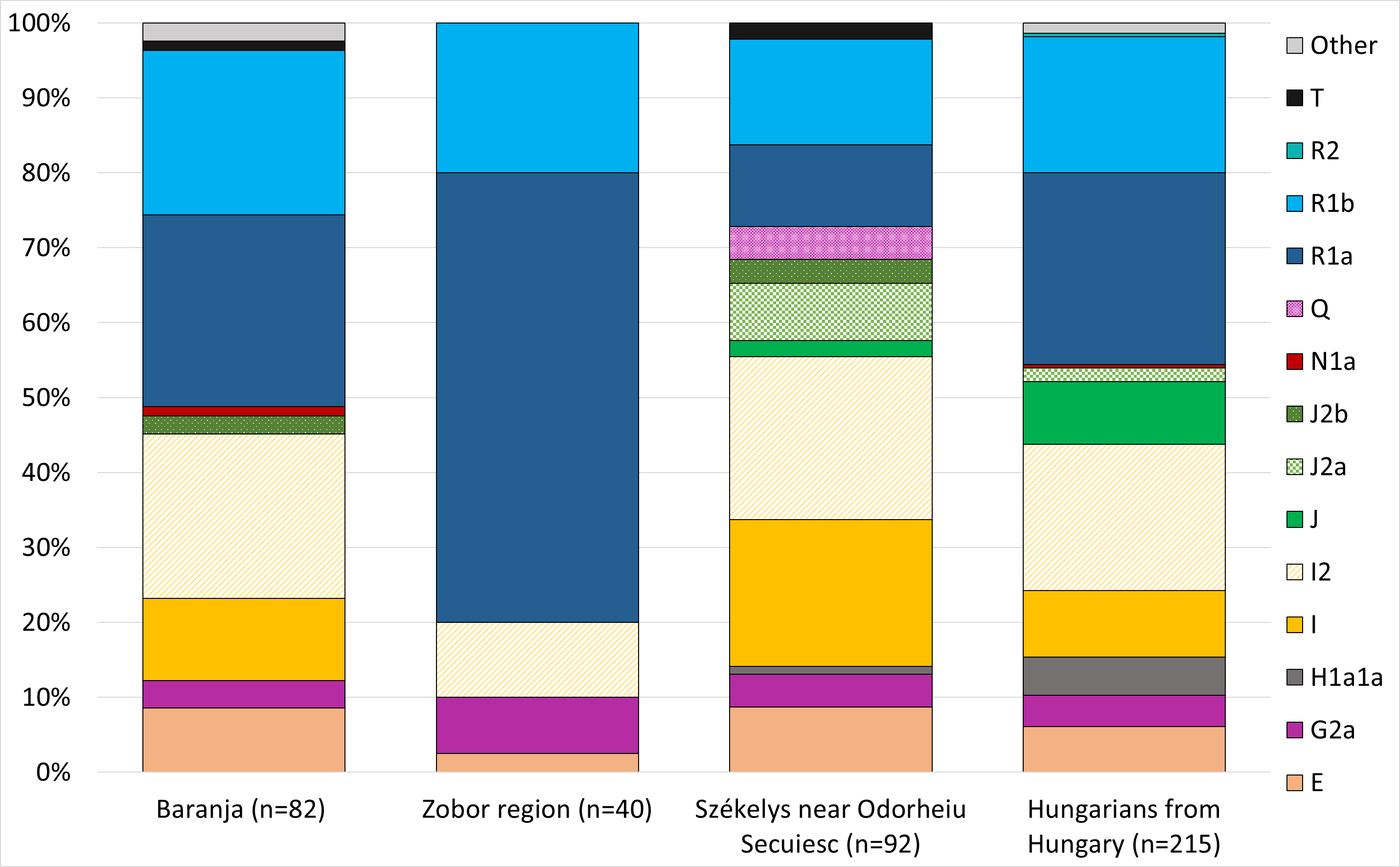
**

**
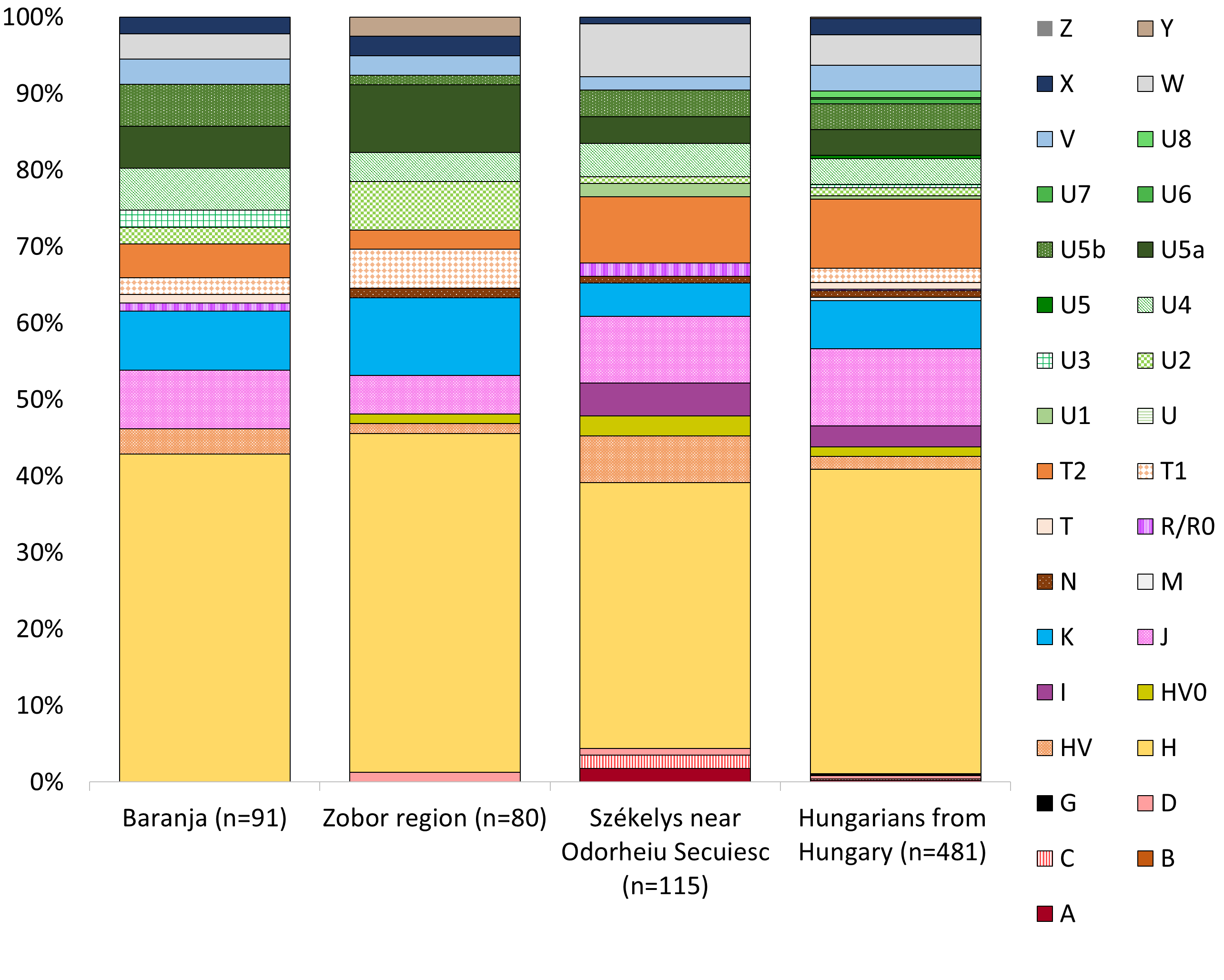
**

**B**

**Fig. S1 Y-chromosomal (Fig. S1 part A) and mitochondrial (Fig. S1 part B) haplogroup frequencies**

Sample sizes are indicated for each group. Differing haplogroup proportions arise, particularly regarding the Y haplogroups of the Zobor region. The majority of paternal and maternal haplogroups has documented East-Central European spectrum (**Table S3**), and less common subhaplogroups and lineages revealed ancient connections to Eastern Eurasian regions. References to previously published uniparental data are presented in **Table S5** and **S7.**

**Haplogroup-based Y-chromosomal analyses**

**
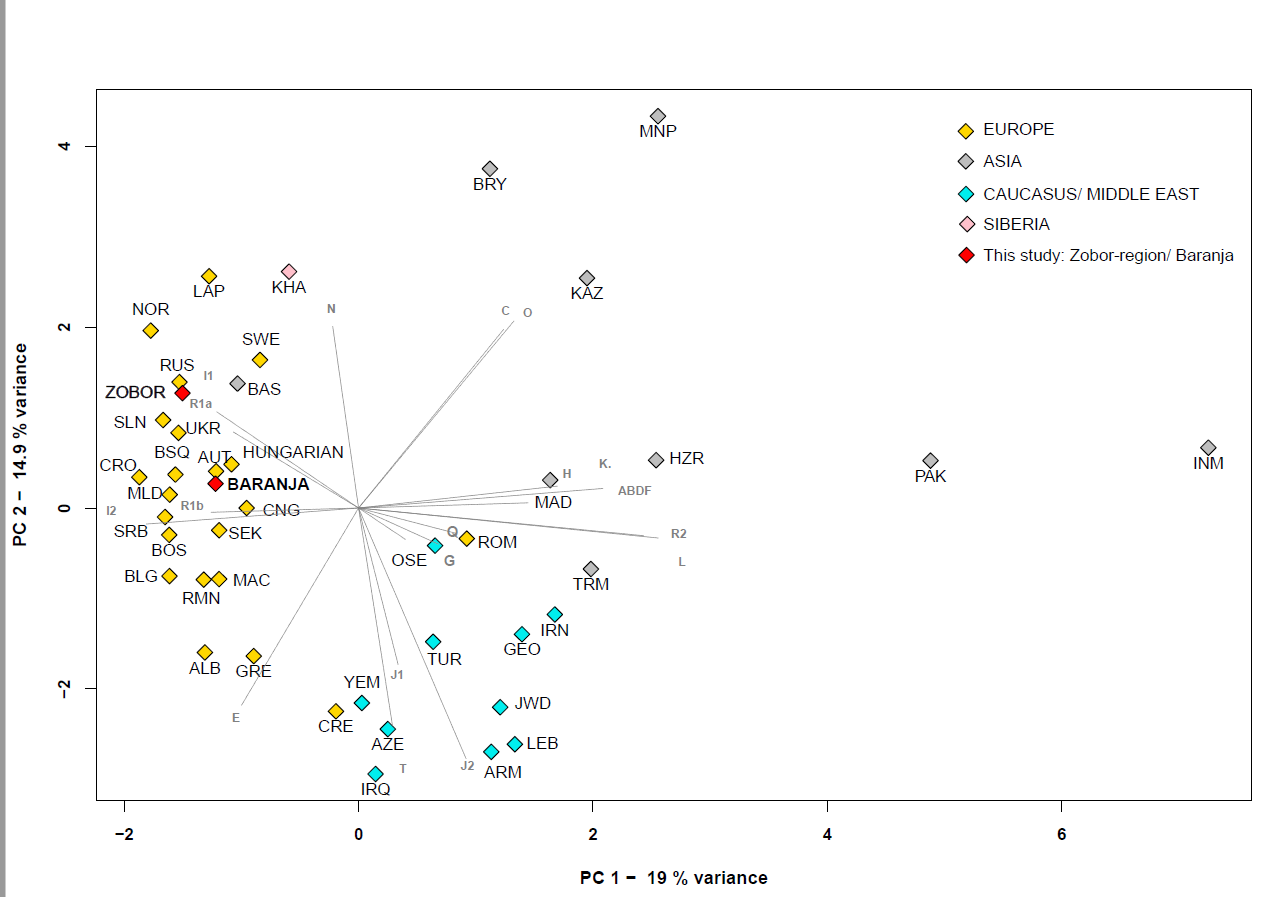
**

**Fig. S2 Principal Component Analysis (PCA) plot of Y chromosome haplogroup profiles**

The PCA shows genetic affinities among 41 populations from Europe, Asia, Caucasus. Original references, number of samples and name codes for all populations included in the analysis and their haplogroup frequencies are listed in Supplementary **Table S7**.

**Y-chromosomal F_ST_ analysis**

We computed Slatkin's linearized F_ST_ distances using Arlequin, treating haplogroup distributions as allele frequency data (see Supplementary **Table S8**). A heatmap, complemented by clustering of pairwise F_ST_ values, was constructed to illustrate the genetic differentiation among the studied populations (**Fig. S3**). The populations from the Zobor region and Baranja cluster together and are closely related to the Slovenian, Hungarian, Moldovan, Ukrainian, and Russian populations. Among them, the Slovenians are the most similar to the Zobor population, while the Baranja group closely aligns with the Hungarian, Moldovan, and Slovenian populations.


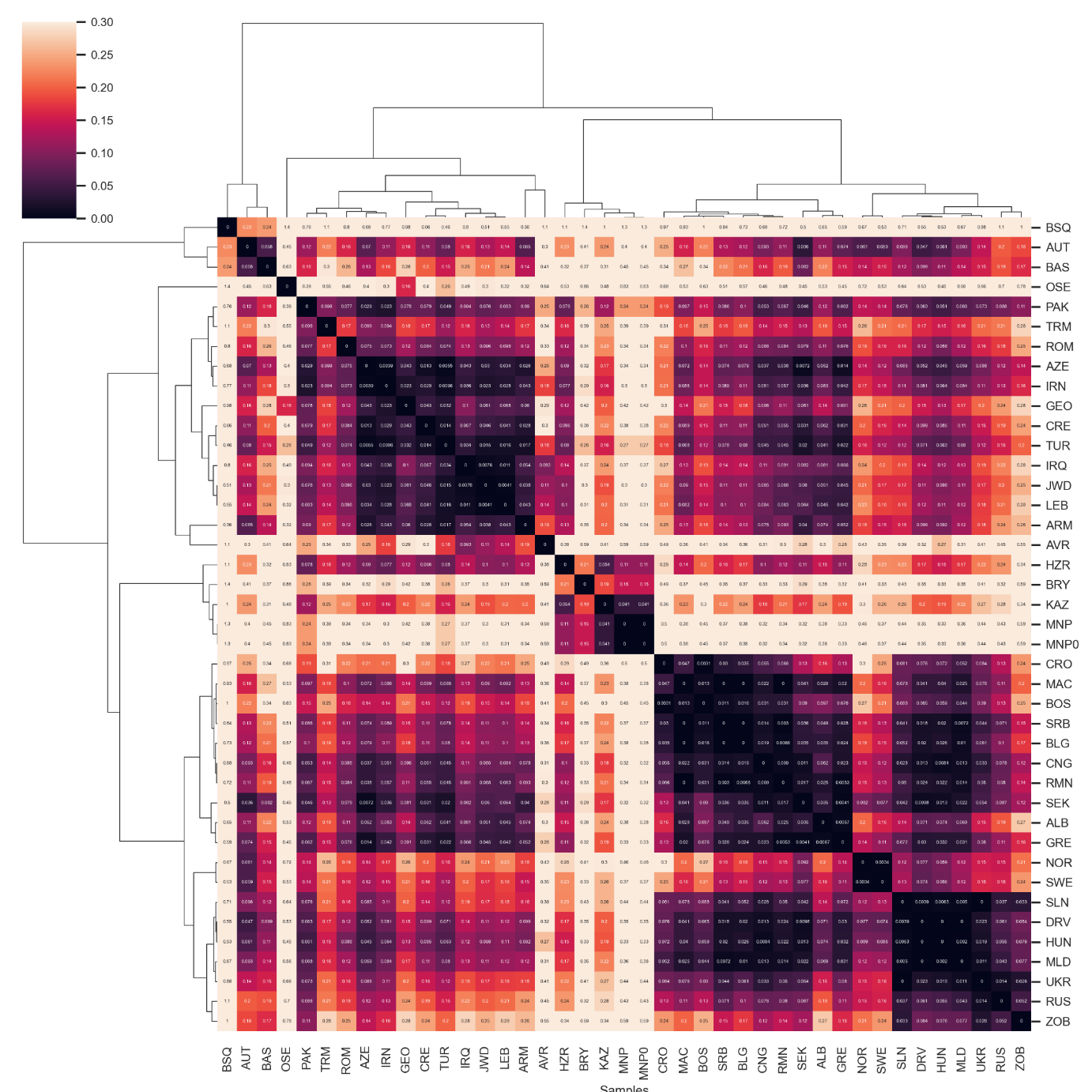


**Fig. S3 Heatmap of pairwise F_ST_ values with clustering (based on Y chromosomal haplogroup frequencies) applied for modern populations (n = 41) from West Eurasia** (color scale ranging from dark purple to yellow). The lighter block colors indicate larger genetic differentiation, whereas the darker colors show closer genetic affinities between the pairs of populations. We calculated the values in Python using the seaborn clustermap function with parameters ‘correlation’ distance metric and ‘complete linkage’ method.

**Y-STR based phylogenetic analyses**

**A**


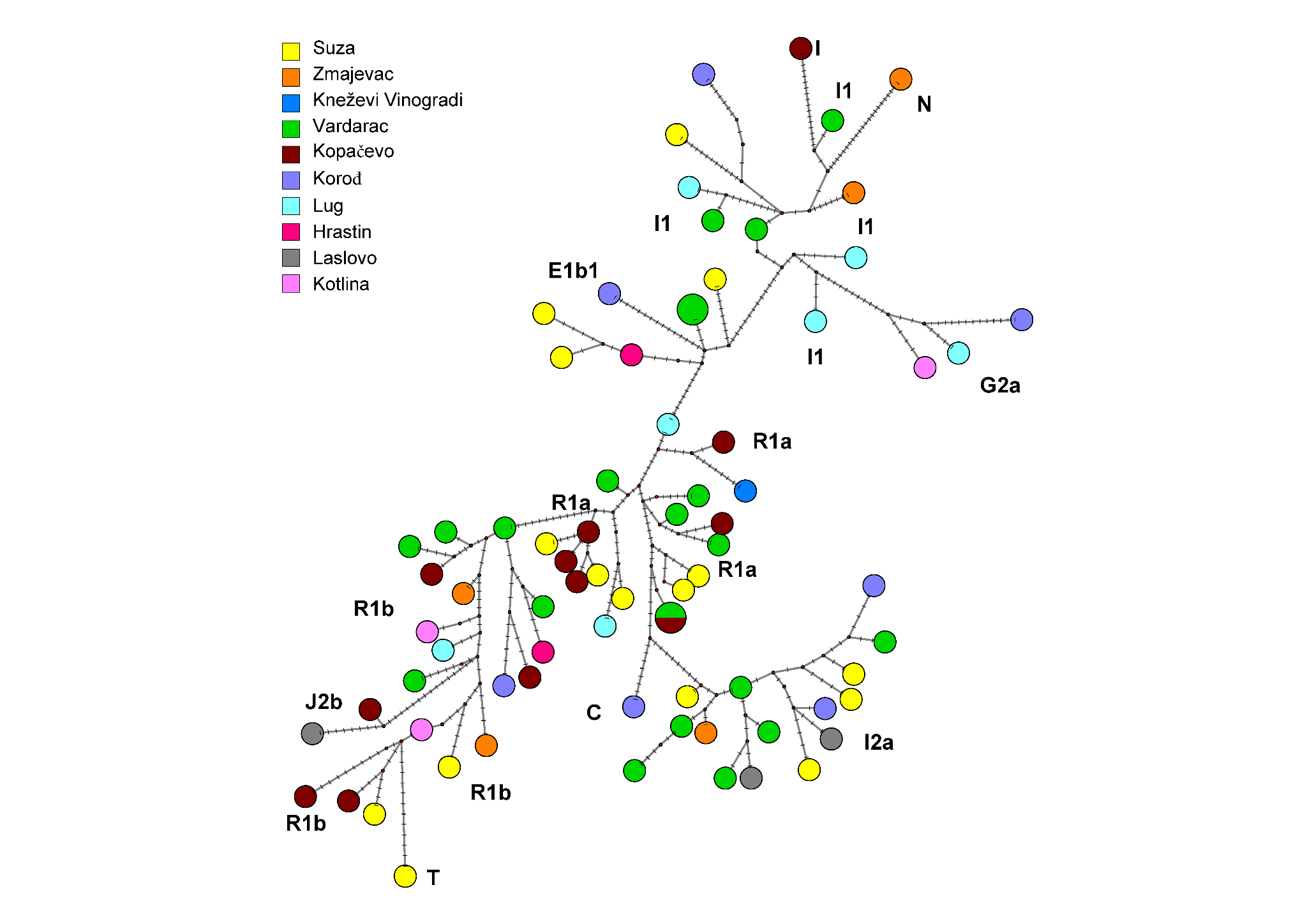

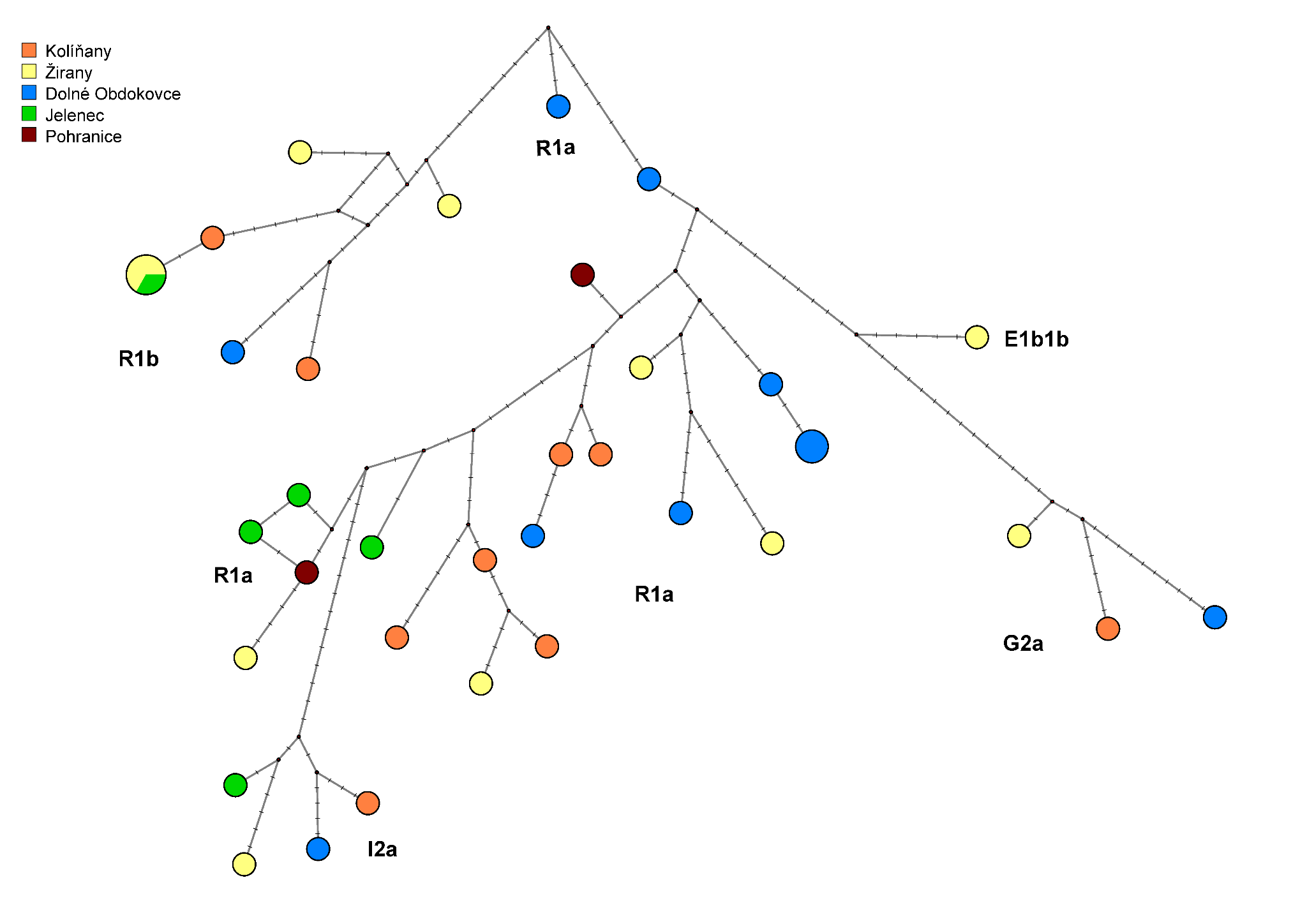


**B**

**Fig. S4 Median-joining networks of the Baranja region (A) and Zobor region (B) Y-STR datasets based on 21 STR data**Colors represent villages as the sources of the samples. The distribution of Y haplogroups between the villages did not show a characteristic pattern or patrilineal system, as the observed haplogroups displayed intermingling across the geographical regions. The smallest circle corresponds to a single haplotype.

**B**

**A**

**
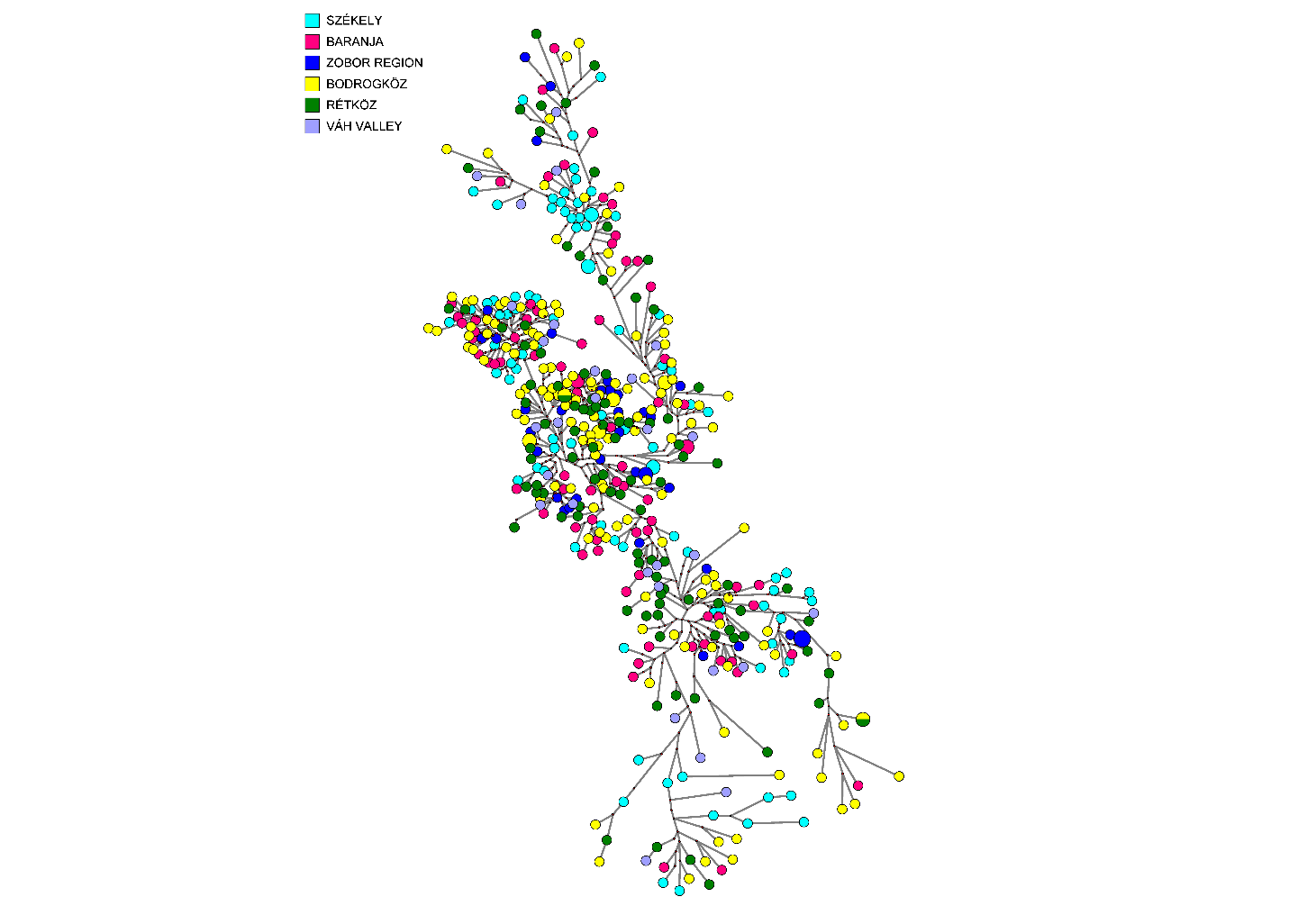

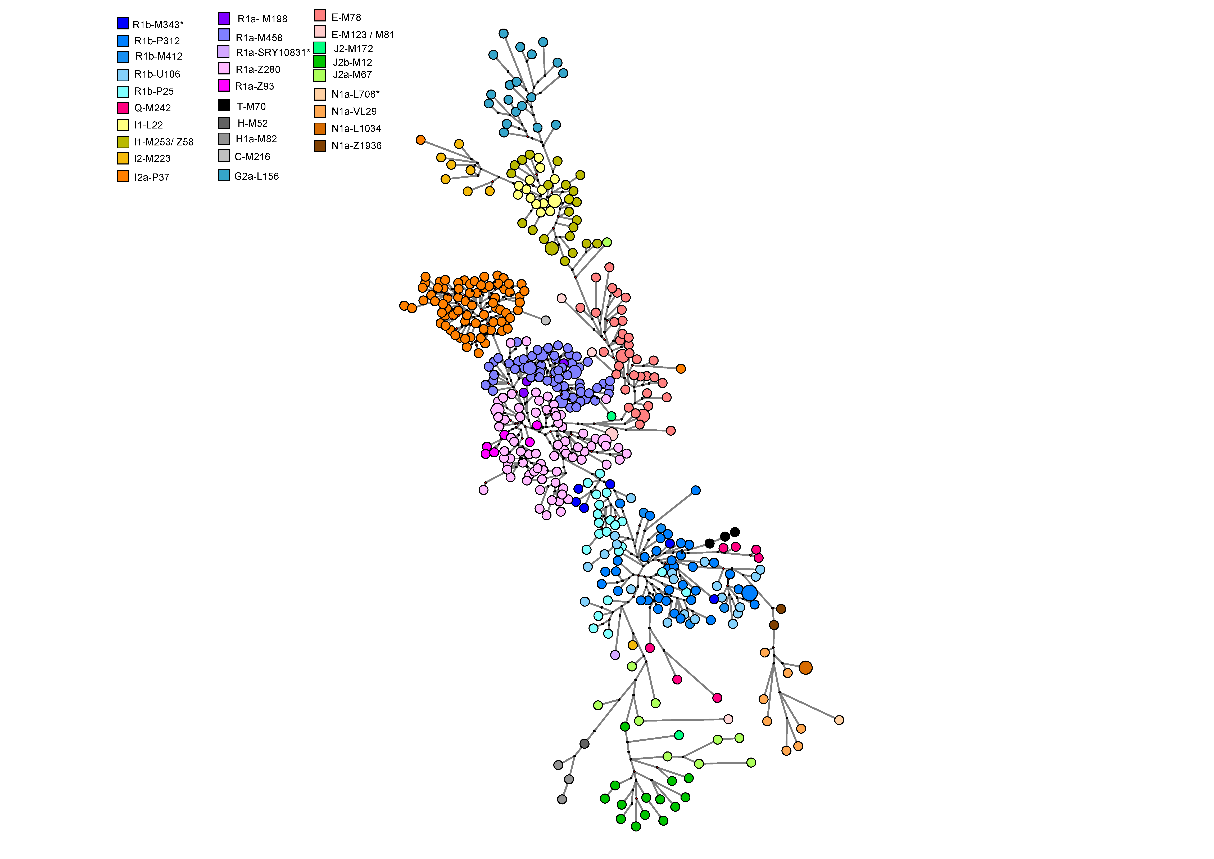
**

**Fig. S5 Summarized MJ Networks of Hungarian-speaking male populations**
Median-Joining Networks of all Hungarian-speaking male populations analyzed from Baranja, Zobor region, Transylvania, Bodrogköz, Rétköz and Váh valley based on 21 STR data ^7–9^ colored by regions (part A) and by haplogroups (part B). The contemporary male population in the Carpathian Basin does not manifest a distinct Y-haplotype structure reflective of its geographical characteristics (see **Fig. S5 part A**), while we can see well-separated clusters according to haplogroups (see **Fig. S5 part B**).

**Median-joining network of 180 N-M46 haplotypes**

Based on 180 haplotypes, an N-M46 (ISOGG 2019-2020: N1a1-M46/Tat) MJ network was constructed using populations previously studied by Bíró et al. (2015); Fehér et al. (2015); Szeifert et al. (2022); Pimenoff et al. (2008); Ilumäe et al. (2016) (**Fig. S6**).

From the perspective of Hungarians, haplotype cluster 3 is pertinent. Cluster 3 comprises three identical haplotypes: two Hungarian and one Northern Mansi, with the Baranja N-M46 haplotype included among them.

As it can be seen from **Fig. S6**, the right branch of the network has almost exclusively representatives of the Finno-Ugric language group, except for Bashkirs. Bashkirs have been living close to the Ugric peoples around the Ural Mountains, where admixture can be traced back for a millennium. This network branch can be derived from cluster 2, where the Buryats form the majority of the haplotypes.

s
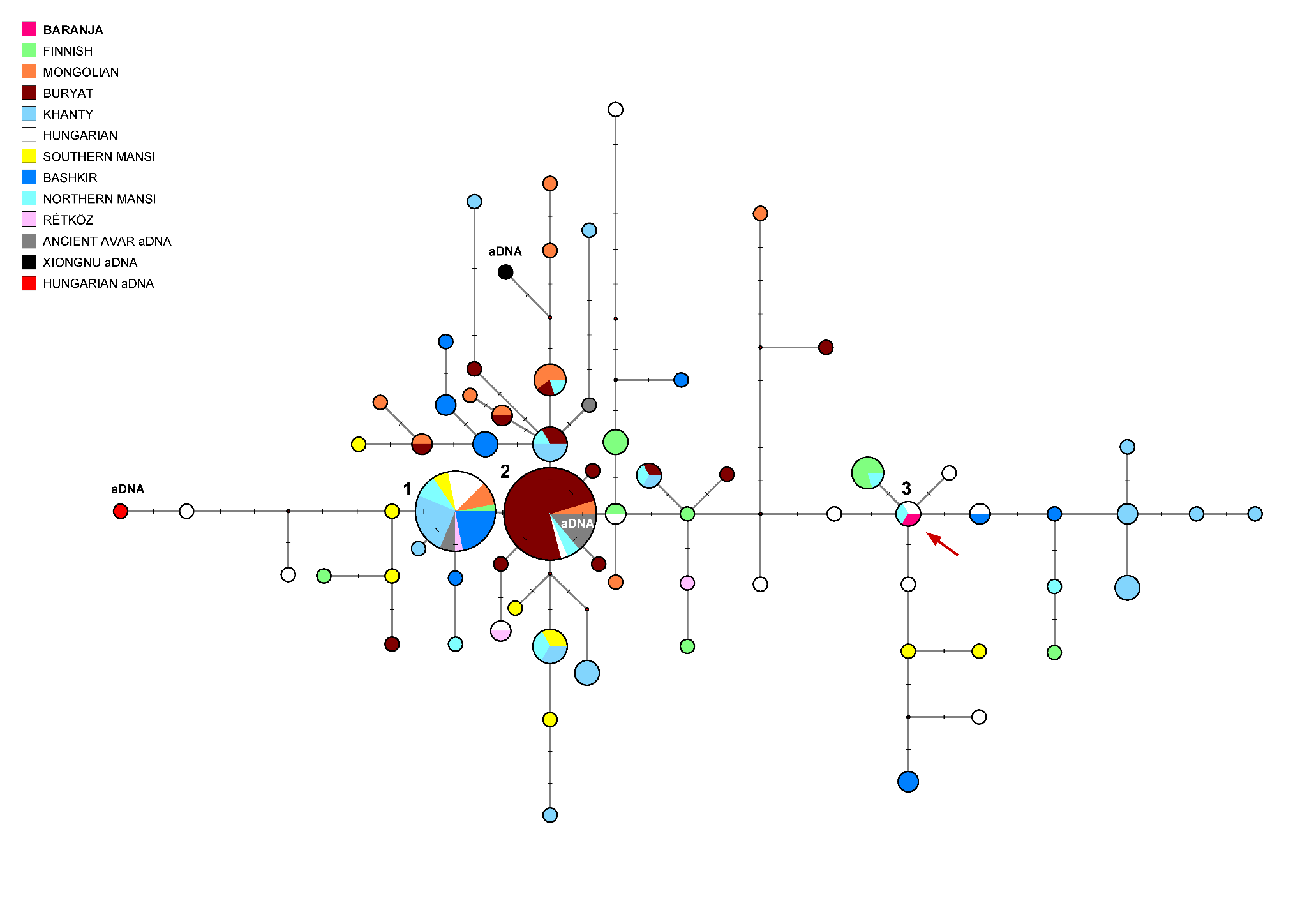


**Fig. S6 Median-Joining Network of 180 N-M46/Tat (N1a1) haplotypes**

The circle sizes are proportional to the haplotype frequencies. The smallest circle is equivalent to one individual. The numbers on the network indicate the haplotype clusters described in the text. One Hungarian aDNA haplotype from Örménykút (Ö52/50, Fóthi et al. (2020)) was positioned six mutational steps away from cluster 1 and formed a haplotype branch with two present-day Hungarian males. Arrows show the samples of this study.

Macrohaplogroup N-M231 is widespread from Scandinavia to the Kamchatka in North Eurasia and it is the most frequent haplogroup in Siberia ^16,17^. Based on the geographic distribution of the parahaplogroup N*-M231, it most likely originated in Southeast Asia, whereas its most widespread subgroup is N-M46 ^17^. Other authors consider Southern Siberia as its geographical origin ^18^.

The present-day Hungarian Y-chromosomal gene pool contains only a small percentage of N-M46 (1%) and has a distribution typical of East-Central Europe ^19^. However, its incidence is higher among the Hungarian-speaking Bodrogköz in East Hungary (6.2%) and among Székelys from Miercurea Ciuc, Romania (6.3%) ^7,10^. According to the results of the Hungarian aDNA studies, N-M46 is detected at a higher frequency (17-36%) in the Hungarian Conquest Period population in the Carpathian Basin ^15,20^.

**Median-joining network of 196 I2a-P37 haplotypes**

The MJ network of 196 I2a-P37 STR haplotypes from 14 populations is illustrated in **Fig. S7**. Greek, Irish ^21^, Catalan samples ^22^, Croatian samples from [FTDNA](https://www.familytreedna.com/groups/i-2a-hap-group/dna-results) ^23^, and Slovakian samples ^24,25^ were used in the analyses. One sample (Karos II, grave 16) from the Hungarian Conquest Period ^15^ and all other samples originated from previously studied and published populations^8–10,26^. In this network, Hungarian speakers residing in rural regions and adjacent countries were distinguished separately. While they are well-documented, there is limited data available that combines both haplotype and haplogroup information from other European populations.


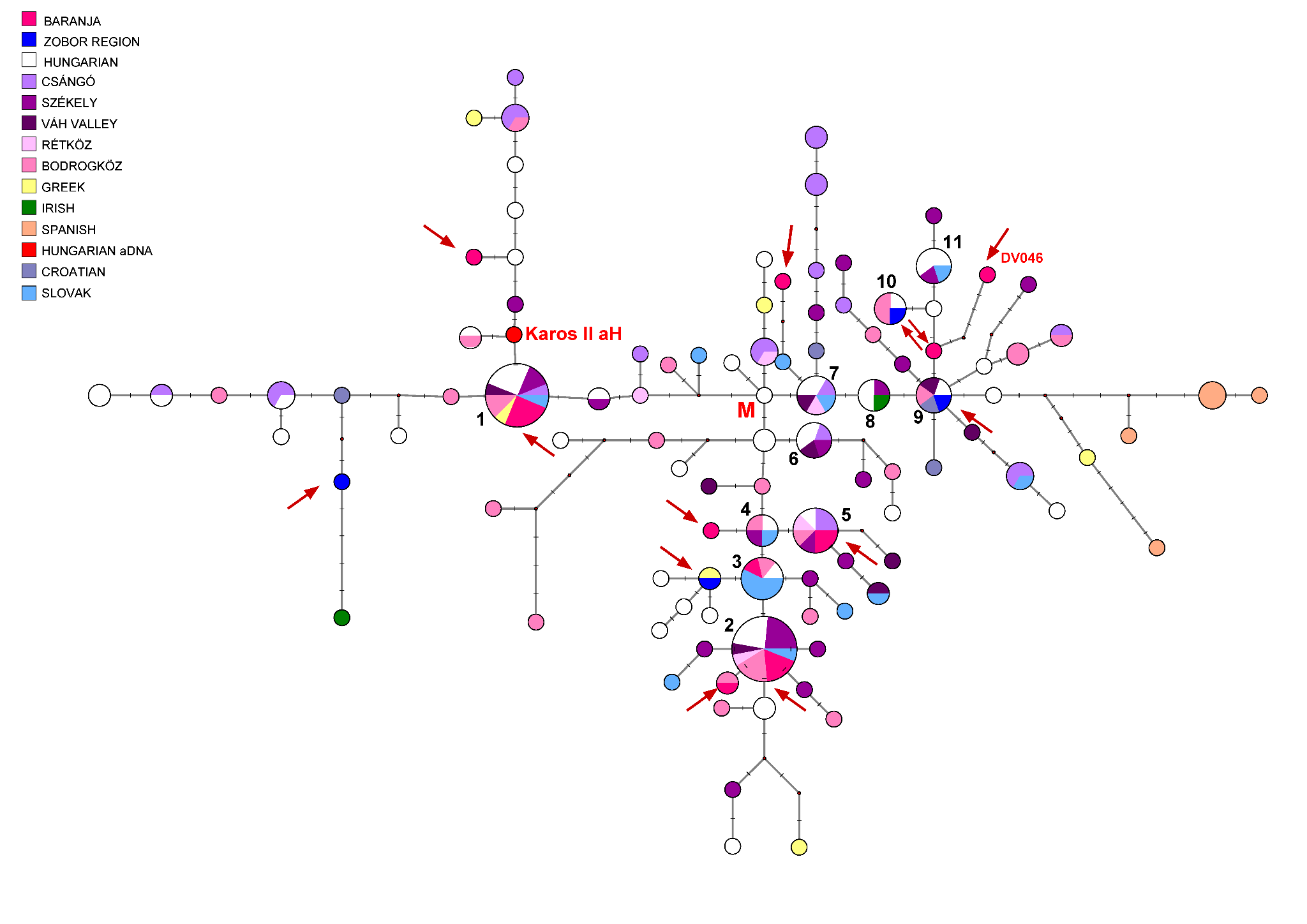


**Fig. S7 Median-Joining Network of 196 I2a-P37 haplotypes**

The circle sizes are proportional to the haplotype frequencies. The smallest circle is equivalent to one individual. The numbers on the network indicate the haplotype clusters. M means the median haplotype. Arrows show the samples of this study.

Haplogroup I-M170 is a fundamental element of the European Y-DNA gene pool, representing, on average, 18% of the total male lineages. Its near absence in other regions, including the Near East, indicates its likely emergence in Europe, potentially before the Last Glacial Maximum (LGM) ^27^.

Haplogroup I-M170 has two major subgroups: I1-M253, which is common in Scandinavia, and I2-M438. I2 subgroup I2a-P37 (formerly I1b, currently I2a1a), extends from the eastern Adriatic to eastern Europe and noticeably decreases towards the southern Balkans. I2a probably diffused from its homeland, Eastern Europe or the Balkans after the LGM ^28^.

In contrast, I2b-M223 (formerly I1c, currently I2a1b1) most likely arose in southern France/Iberia and similarly to the other subclades, it underwent a postglacial expansion ^28^. Taken together, these observations suggest that haplogroup I-M170 may have played a central role in the process of human recolonization of Europe from isolated refugia after the LGM and suggest that a comprehensive phylogeographic study should localize the *in situ* origin and spread of major male founders ^28^. According to the study of Peričić et al. (2005), the I2a haplogroup corresponds to the historic expansion of the Slavs that may have taken place in the middle of the first millennium AD and resulted in significant admixture with the substratum populations living in Eastern Europe. Their haplogroup, subgroup I2a, is widespread among the Slavs, especially the western south Slavs, but has also been detected up to 3-7.1% in populations of the Northern Caucasus ^28^.

Based on our network, there are admixtures and shared lineages between the Hungarian-speaking populations (clusters 1-11), and also with that of the neighbouring Slavic countries (clusters 1-4 and 7-8), included in the study (**Fig. S7**). Among the conqueror Hungarians, there were three samples that had haplogroup I2a, but due to the lack of overlapping STR loci, only one sample could be included in the study. According to the authors, these three I2a samples were close relatives on the paternal lineage ^15^.

Based on the YSEQ I2 panel, the deeper classification of the DV046 (Baranja) sample is I2-Y125026, which is I2a1b2a2b2b~ according to ISOGG 2019-2020. Modern-day Yfull data from this haplogroup are known from Hungary, Croatia, Romania, Serbia, Turkey, Montenegro, Greece, Poland, and Russia. As P37 subgroup has been prevalent in the Southeastern European area already at the time of the Hungarian conquest, and is rare/absent in the Volga-Ural Hungarian-related communities^12^, we conclude that it most probably represents a local lineage in the modern Hungarian-speaking populations, as it was similarly an European component in the mixed population of the Conquest period.

**Median-joining network of 280 G2-L156 Y-STR haplotypes**

The MJ network of 280 G2-L156 haplotypes is depicted in **Fig. S8***.* The samples belong to three identifiable subgroups (L497 (G2a2b2a1a1b in ISOGG 2019-2020) within P303, M406 (G2a2b1 in ISOGG 2019-2020), L91 (G2a2a1a2 in ISOGG 2019-2020)) within haplogroup G2-L156. The haplotype cluster 1 is shared by nine populations, including four Hungarians (Hungarian from Hungary, Bodrogköz, Rétköz and Zobor region) and further males from Germany, Austria, Italy, and the Caucasus. The haplotype cluster 2 is shared by three populations (cluster 2 in **Fig. S8**), important from the Baranja and Zobor region aspects. Haplotype cluster 3 is shared by three populations and it is to be the founding haplotype of subhaplogroup of L497. L497 can be derived from the Early Neolithics ^30^, and is still frequent in Tyrol nowadays ^31^. Based on the pattern of the network, it can generally be said that almost all Hungarian males including samples from the present study are grouped either with Caucasian population samples such as Balkarians/Karachays or with the Tyrolean (see M497, M406 branches) and some German samples. Most Ossetian, Lezgian and Abkhazian males from the Caucasus form independent haplotype clusters, except for the Circassians, which are scattered within the network.

The age of accumulated STR variation within the G2-L156 lineage for 280 samples is estimated to be 20.5±4.5 kya (95% CI=16.0-25.0 kya), considering the haplotype belonging to cluster 1 is the founder one. This value is nearly identical to its sequence-based calculation of 18.2 kya. The estimated age for the L497 branch is 8.1±2.3 kya (95% CI=5.8-10.4 kya) (**Fig. S8**).


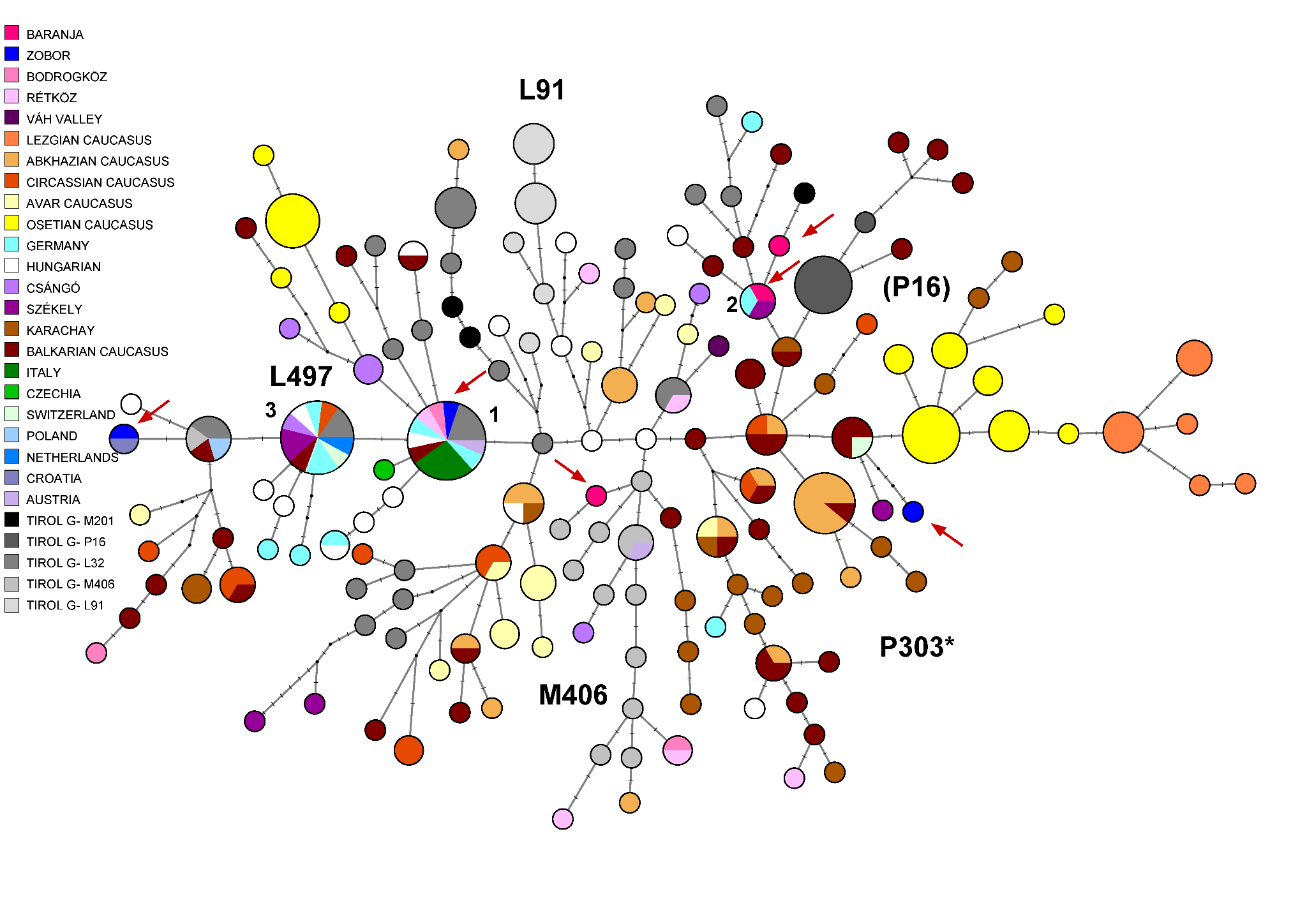


**Fig. S8 Median-Joining Network of 280 G2-L156 haplotypes**

The circle sizes are proportional to the haplotype frequencies. The smallest circle corresponds to one sample/individual.

According to current knowledge haplogroup G is associated with the spread of agriculture from Fertile Crescent, particularly in Europe. Haplogroup G was first discovered in Europe and Georgia ^27^ and was later tested in Caucasian and Hungarian populations ^19,32^. The frequency of subhaplogroup G2a-P15/L156 is about 4% in the Hungarian population ^19^, 6% of the Csángó population (Hungarian minority) in Romania, and 4% of the Hungarian minority in the Székely population, Romania ^10^. Because the frequency of the haplogroup is low in Hungarian-speakers, only the L156 was tested from the downstream SNPs, which is phylogenetically equivalent to SNP P287 marker (G2 haplogroup) ^33^. Rootsi et al. (2012) analyzed 113 Hungarian males, of which 2 belonged to basal haplogroup G-M201 (1.8%) and to subgroups G2a-L497 (0.9%) and G2a-M406 (0.9%). According to the authors, P303 SNP represents the most common and widespread G subgroup (G2a2b2a on ISOGG 2019-2020), whereas G2a2b2a1a1b L497 lineages occur in Europe. The highest frequency of G2a-P303 is detected in populations from the Caucasus, specifically among South Caucasian Abkhazians (24%), Northwest Caucasian Adyghe (39.7%), and Cherkessians (36.5%) ^30^. Another common subclade is M406 (G2a2b1). The peak frequency G2a-M406 is in the Mediterranean and Central Anatolian (6-7%), as well as in Greek (4%) and Italian (3%) populations. This subgroup was not detected in many other regions where the frequency of P303 is high ^30^. The G2a-P16 lineage is specific to the Caucasus, accounts for one-third of the Caucasian male gene pool and occurs with the highest frequency among North Ossetians (63.6%). Outside the Caucasus Mountain the P16 lineage is present in less than 1% ^30^.

Based on our network analysis, we can also distinguish four subclades: L497, P303, M406, and P16, since the samples with known subhaplogroups in the network are clustered on the same branches (**Fig. S8**). The Hungarians are included in subclades L497, P303, and M406, except for P16, where only three Csángó samples are found, which indicates that the gene flow from the P16 subclade of the Caucasus has been negligible. It needs to be noted, that P16 is not included in the latest Y trees, as it turned out to be a palindromic position. Therefore, deeper typing of these P16 data is necessary.

We included six G2a-L156 samples from the Baranja and Zobor region populations, each of which falls into these three subgroups. Four of them belong to the M406 branch, one Zobor region sample is identical to the assumed modal haplotype. The results indicate a close genetic relationship between the Hungarian-Balkar/Karachay and Tyrolean males, as they share a common haplotype or are grouped into nearly identical haplotypes for one or two mutational steps.

The G2a-L156 haplogroup was also found among the results of aDNA tests carried out in the Carpathian Basin. The researchers showed that one ancient Avar and four Hungarian conqueror samples belonged to the G2a-L293 subgroup, two Hungarian conqueror samples belonged to the G2a-U1 subgroup (G2a2b2a1a1a1), and one Hungarian conqueror sample belonged to the G2a-L30 subgroup (G2a2b) ^15,34^. The U1 and L497 SNPs define subclades within P303 G2a-L30 appeared already in the early farming period in the Carpathian Basin ^35^, whereas its spread to the Caucasus is untackled yet.

**Median-joining network of 126 R1a-Z280 Y-STR haplotypes**

A MJ network of R1a-Z280 (R1a1a1b1a2) samples was generated using 126 haplotypes from nine populations, including 25 samples from the present study (10 Zobor region and 15 Baranja) (**Fig. S9**). All samples included in the network were tested by us and previously published ^10,36^, and no other published Z280 haplotypes and haplogroups are currently available. The founding haplotype of R1a-Z280 was shared by seven samples from three populations (three Bashkirian Mari; three Hungarian and one Zobor region, Slovakia) as presented in **Fig. S9** (Haplotype 1). Further 11 haplotypes were shared by males from different populations. Six of them contained samples from the study regions. All other haplotypes were scattered in the network. The age of accumulated STR variation within R1a-Z280 lineage for 126 samples is estimated to be 15.4±1.0 kya (95% CI=14.4-16.4 kya) considering haplotype 1 to be the founder, which is older than its sequence-based calculation of 4.6 kya ^37^.


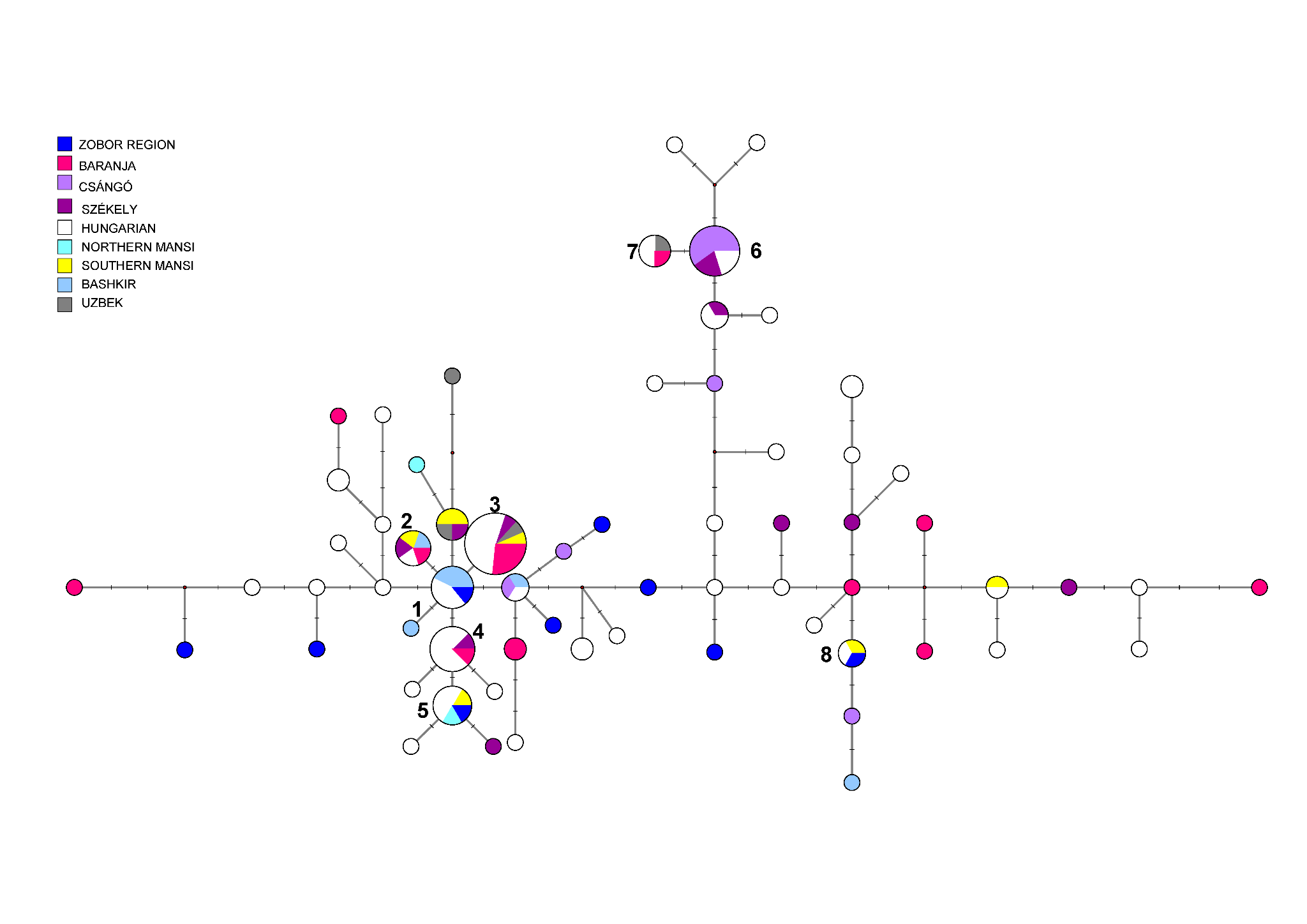


**Fig. S9 Median-Joining Network of 126 R1a-Z280 haplotypes**

The circle sizes are proportional to the haplotype frequencies. The smallest area is equivalent to one individual. A MJ network of R1a-Z280 (R1a1a1b1a2) samples was generated using 126 haplotypes from nine populations, including 25 samples from the present study (10 Zobor region and 15 Baranja). As illustrated in the figure, the R1a-Z280 MJ network presents a notable pattern. The populations examined in this study share common haplotypes with not only Hungarian-speakers but also Finno-Ugric-speakers (haplotypes 1-3, 5, and 8). Intriguingly, samples from either the Baranja or Zobor regions also possess shared haplotypes with Uzbek samples (haplotypes 3 and 7). This suggests a deep-rooted common paternal genetic lineage, given the vast geographical distance separating these populations for at least a millennium.

Haplogroups of R1a have been considered as Indo-European migration markers ^38^. R1a-M198 (R1a1a) has three downstream subclades, L664 (R1a1a1a1, North-Western branch), Z283 (R1a1a1b, Eurasian branch), and Z93 (R1a1a1b2, South-Eastern branch), split from their common European ancestor at about the same time, around 6000 - 4000 ybp ^37^. L664 apparently stayed in North-Western Europe; its lineage recovered and began expanding ~4575 ybp. The Z93 subclade began to expand during the Middle-Late Bronze Age migrations to India and the Middle East in the 2nd millennia BC. The Z283 subclade split ~5500 ybp into three branches ^39^. One of them, Z280 (the Central Eurasian branch) moved east to the Russian Plain in 4800 - 4600 ybp and formed at least 16 subbranches there and during the later westward repopulation of Europe in the first millennium BC—first millennium AD. As seen in **Fig. S9**, the MJ network of R1a-Z280 reveals an interesting pattern. Both populations investigated in the present study shared common haplotypes with not only Hungarian-speakers, but Finno-Ugric-speakers (Haplotypes 1-3, 5 and 8). Interestingly, the Baranja or Zobor region samples also shared common haplotypes with Uzbek samples (Haplotypes 3 and 7), implying a common deep paternal genetic ancestry, since these populations were separated by thousands of kilometers for at least thousand years ^40^.

According to our hypothesis, R1a-Z280 could have entered the gene pool of the Ugric peoples (Khanty and Mansi) in the Russian Plain, including the Volga Uplands and the North Pontic steppe region. People from this area could have brought the Z280 marker to the Carpathian Basin. So far, the R1a-Z280 marker has not been detected among the results of the bone samples from the Huns, Avars and conquering Hungarians, but this might change with increasing future testing. To clarify this issue, more populations from as many geographic regions as possible must be studied using downstream SNPs and/or the entire Y-chromosome sequencing.

**Median-joining network of 195 J2b-M102/M12 Y-STR haplotypes**

We created an MJ network of 195 J2b-M102/M12 haplotypes from 12 populations. Except for the Hungarian-speakers and Uzbek populations we examined, all population samples originated from the FTDNA database. The two biggest clusters are shared by 16 (cluster 1) and 15 (cluster 2) males from eight and seven populations, respectively (**Fig. S10)**.

Several other smaller haplotype clusters show different admixtures or common origin of Y-chromosomal lineages of Hungarian-speakers investigated in the study (see arrows on **Fig. S10**). Circles with mixed colors such as Hungarian-German, Hungarian-Balkan, Hungarian-Russian and Polish-Hungarian can be observed.

The age of accumulated STR variation within the J2b-M102 lineage for 195 samples is estimated to be 27.4±1.5 kya (95% CI=25.9-28.9 kya), considering the median haplotype (see cluster 1 in **Fig. S10**) is the founder, which is much higher than its sequence-based calculation of 15.8 kya ^37^.


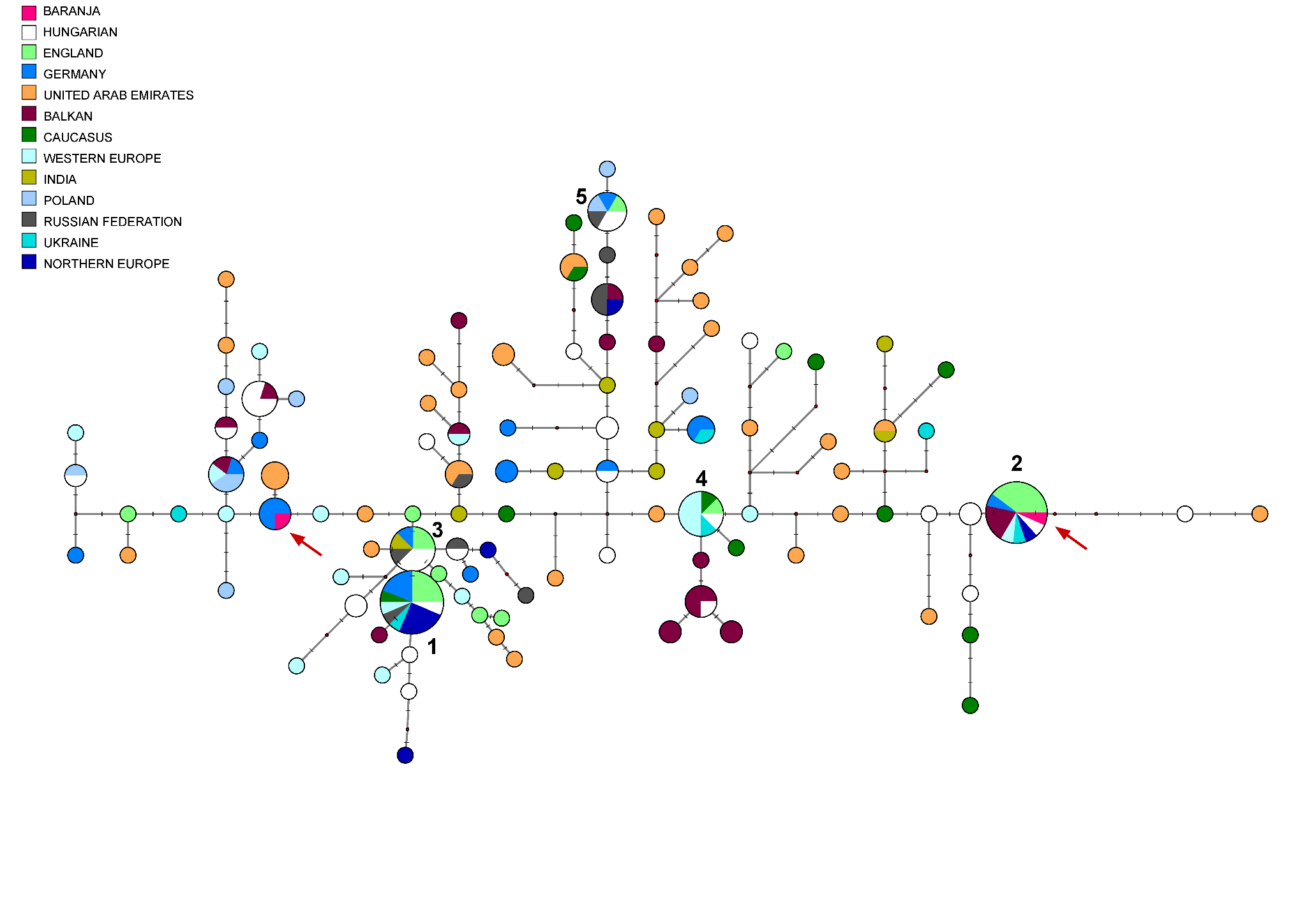


**Fig. S10 The Median-Joining Networks of 195 J2b-M102/M12 haplotypes**

The circle sizes are proportional to the haplotype frequencies. The smallest circle is equivalent to one individual. The samples were pooled as followings: Northern European (NE): Norwegian, Dutch, Swedish, Belgian, and Finnish populations; Western European (WE): French, Italian, Spanish, and Portuguese populations; Southeastern Europe: Croatian, Serbian, Greek, Bulgarian, Albanian, Kosovan and Macedonian populations; Caucasian: Armenian, Georgian, and Azeri populations; Arabian: Lebanese, Palestinian, Saudi Arabians, Iraqi, Qatari, Omani, Yemeni, Egyptian, Syrian, Algerian, Bahrain, Libyan and Turkic populations. The median-joining network of J2b-M102/M12 haplotypes was constructed from 12 populations. Predominantly, European populations share the same haplotypes (clusters 1-2 and 4-5) in this network, suggesting that the ancestors' admixture occurred in Europe. An exception is the presence of an Indian sample in haplotype cluster 3.

Haplogroup J2-M172 has been associated with the Neolithic spread of agriculture, especially based on modern European data ^27^. Ancient data however rather attests its presence in the region of Iran and the Caucasus in the Neolithic times. A subgroup of J2, J2b-M12/M102, detected from India to Europe, shows very high Y-STR diversity and splits into mainly J2b-M205, frequent in Southern Levant and J2b-M241, most frequent in Greece and the Balkans ^41^. The J2b-M241 subclade has low diversity in the Balkans, indicating different demographic histories.

Based on our observation, haplogroup J2b-M12 was more likely to be found in Hungarian-speaking minorities (Székely, Csángó and Baranja) living in countries surrounding Hungary or in more isolated regions of Hungary (Bodrogköz, Rétköz), with a frequency of 3-5%.

Based on **Fig. S10**, almost only the European populations share the same haplotypes (clusters 1-2 and 4-5), indicating the admixture or common origin, except that there is an Indian sample in the haplotype cluster 3. Arabian and Indian samples are mainly scattered across the network. It cannot be deduced from the pattern of the network, how well the subgroups of J2b (J2b2a-M241 and J2b1-M205) are separated from each other. Furthermore, the populations are not separated geographically either, since individuals from Arabian populations, for example, occur in almost all branches. Presumably, these Arabian population samples would belong to subclade J2b-M205, as Battaglia et al. (2009) ^41^ detected. Individuals from the Indian population are mainly clustered in the center of the figure (see dark olive circles in Figure **S7**). According to a study by Singh et al. (2016) ^42^, J2b-M102/M12 in India consists largely of J2b-M241 chromosomes, while in the northwestern (NW) country, J2b1-M205 is present to a small extent. These observations can also be reflected in our network.

According to a recent study based on Y chromosome sequencing results, ^43^ observed that J2b-M12 did not spread continuously. The conclusion of the study is that the phylogeography of haplogroup J is complex and hardly explained by the presence of a single population harboring the major lineages at the onset of agriculture and spreading westward, but rather date to the Bronze Age ^43^. This aligns with the aDNA datasets, where J2b first emerged in the Bronze Age of Southeastern Europe and was not prevalent in the Neolithic era of the Balkans ^44^.

**Median-joining network of 279 C2-M217 Y-STR haplotypes**

**Fig. S11** depicts the MJ network of 279 C2-M217 haplotypes from 15 populations. Korean, Hazara, Manchu, Japanese, Kalmyk, and Kazakh population samples were used from Wei et al. (2018) ^45^. All other samples from populations were published in ^10^.

The biggest haplotype cluster is shared by 40 males from six populations (cluster 1). Haplotype cluster 1 is likely to be the core haplotype of C2-M217 haplogroup. Some other population samples also form an independent homogeneous haplotype cluster, such as Kazakh Madjars, Buryats, Hazaras, Japanese or Koreans. Mainly Korean and Japanese chromosomes were scattered in the middle of the network. The interesting phenomenon is that a Baranja C2-M217 sample is located on the same branch as Koreans and Hazaras.

The age of accumulated STR variation within the C2-M217 lineage for 279 samples is estimated to be 28.3±5.8 kya (95% CI=22.5-34.1 kya), considering the core haplotype (see 1 in **Fig. S11**) is the founding, where the upper limit of the confidence interval is consistent with the sequence-based calculation of 34 kya ^37^.


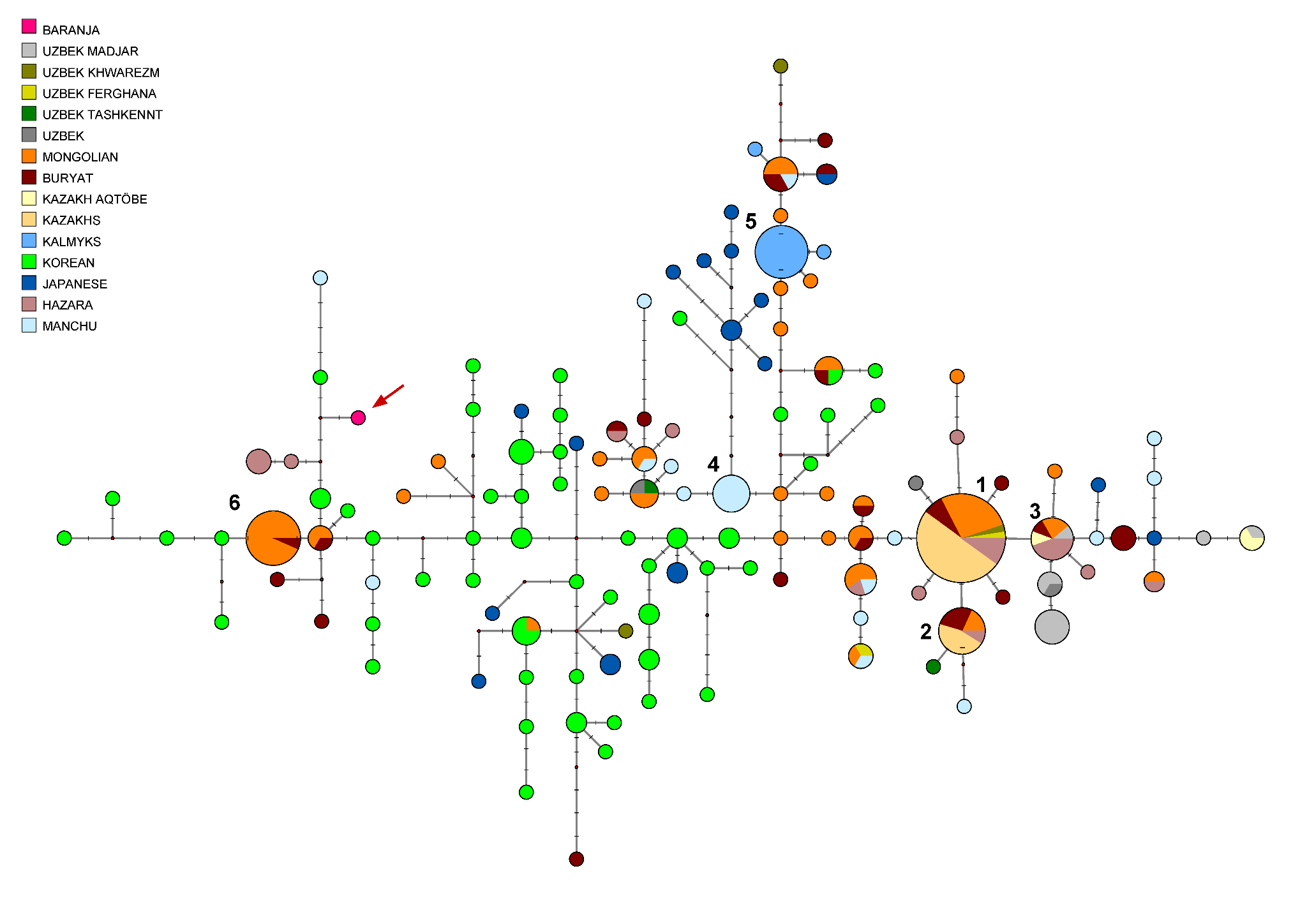


**Fig. S11 The Median-Joining Network of 279 C2-M217 haplotypes**
The circle sizes are proportional to the haplotype frequencies. The smallest area is equivalent to one individual. Median-joining network of C2-M217 haplotypes was based on 15 populations. Several haplotype clusters can be seen in the figure, which shows different admixtures of Asian populations. Our Baranja Hungarian sample from Croatia is located on a branch with Hazara, Korean and Manchu samples far from the star-cluster. This branch can be derived from a haplotype consisting of two Mongolian and one Buryat samples.

Haplogroup C2-M217 (previously C3), is the most frequent haplogroup branch of C-M130 and found mostly in Central Asia, Eastern Siberia, and significant frequencies in parts of East Asia and Southeast Asia including some populations in the Caucasus, Middle East, South Asia, East Europe ^46,47^. There is a well-known haplotype pattern within haplogroup C2-M217 that was first discovered and called for the star-cluster (C2*-Star Cluster) in Zerjal et al. ^46^. This haplotype cluster had two characteristic features: (1) there was a high frequency of a cluster of closely related lineages; (2) star-cluster chromosomes were found in 16 populations throughout a large geographical area extending from Central Asia to the Pacific, thus, they do not result from an event specific to any single population. The authors estimated ∼1,000 years for the TMRCA (95% CI ∼700–1,300 years). Based on their calculation, the origin was most likely in Mongolia, where the largest number of different star-cluster haplotypes is found. Thus, a single male lineage, probably originating in Mongolia, has spread in the last ∼1,000 years to represent ∼8% of the males in a region stretching from northeast China to Uzbekistan. According to the authors, this chromosome is present in about 16 million males, or 0.5% of the world's total population, and their available evidence suggested that Genghis Khan carried it. The boundary of the Mongol Empire, which corresponded to the borders of the regions controlled by the later Mongol Khans, closely matched the distribution of the star-cluster chromosomes, along with the Hazaras. The Hazaras of Pakistan are of Mongolian descent, and many consider themselves to be direct male descendants of Genghis Khan. By documenting genealogy, these relationships were constructed from their oral histories. Most Hazara Y-STR haplotypes were indeed found in the star-cluster, which is otherwise not detected in Pakistan, supporting their own oral tradition, and suggesting that Genghis Khan carried (probably his soldiers) the star-cluster haplotype ^46^.

As depicted in ***Fig. S11***, a distinct star-cluster (cluster 1) is evident where 40 males from six populations share the same haplotype: Mongolian, Buryat, Hazara, Kazakh, and two Uzbek populations. According to a recently published paper based on whole genome sequencing results, the star-cluster, or chromosomes close to it, were found in Uzbek, Nogay, Afghan Hazara, Chinese Dauer, Manchu and Kazakh populations ^45^. These published results are fully consistent with our observations and overlap and confirm the result of the present study.

Our Baranja Hungarian sample in Croatia (see arrow on ***Fig. S11***), whose origin we were looking for, is located on a branch with Hazara, Korean and Manchu samples far from the star-cluster. This branch can be derived from a haplotype consisting of two Mongolian and one Buryat samples. As another data concerning the recent Hungarians, it can be mentioned that the C2-M217 haplogroup was detected in 0.2% of the Hungarian samples submitted to the FTDNA database, which means that, although rarely, the C2-M217 haplogroup is present among Hungarian speakers.

According to aDNA study, only one C2-M217 sample was found, which had a double allele at the DYS19 locus, among the results of the conquering Hungarians ^15^. Based on that, it could be classified in the M86 subgroup of C2-M217 (C2a1a2a). According to another Hungarian aDNA study, C2-M217 samples were also found among early Avar, middle and late Avar period bone samples in the Carpathian Basin ^34^. Since haplotype data were not available for these samples, we could not include them in the network.

**Median-joining network of 370 N-M46 Y STR haplotypes**

We also constructed the N-M46 network using 15 Y-STRs, incorporating additional ancient samples related to ancient Hungarians from the Ural and Volga regions (**Fig. S12**). The Baranja sample diverges from a Bashkir haplotype by two mutational steps, while the other Hungarian males share their haplotype with medieval samples from the Volga and Ural regions. The closest cluster to the Baranja sample is cluster 1 which shares more samples from different populations. It contains haplotypes from Gulyukovo, and also more early Hungarian samples from Karanajevo, Gornovo and Uyelgi can be found in the nearby cluster 2 (**Fig. S12**). The Avar period samples belong to a different subgroup (N1a1a1a1a3a-F4205) of the N-M46 clade, as corroborated by genomic studies ^48,49^. Although substructures observed on the 15 Y-STR network, we conclude that this lineage in the Baranja population has a Conquest Period origin in the Carpathian Basin.


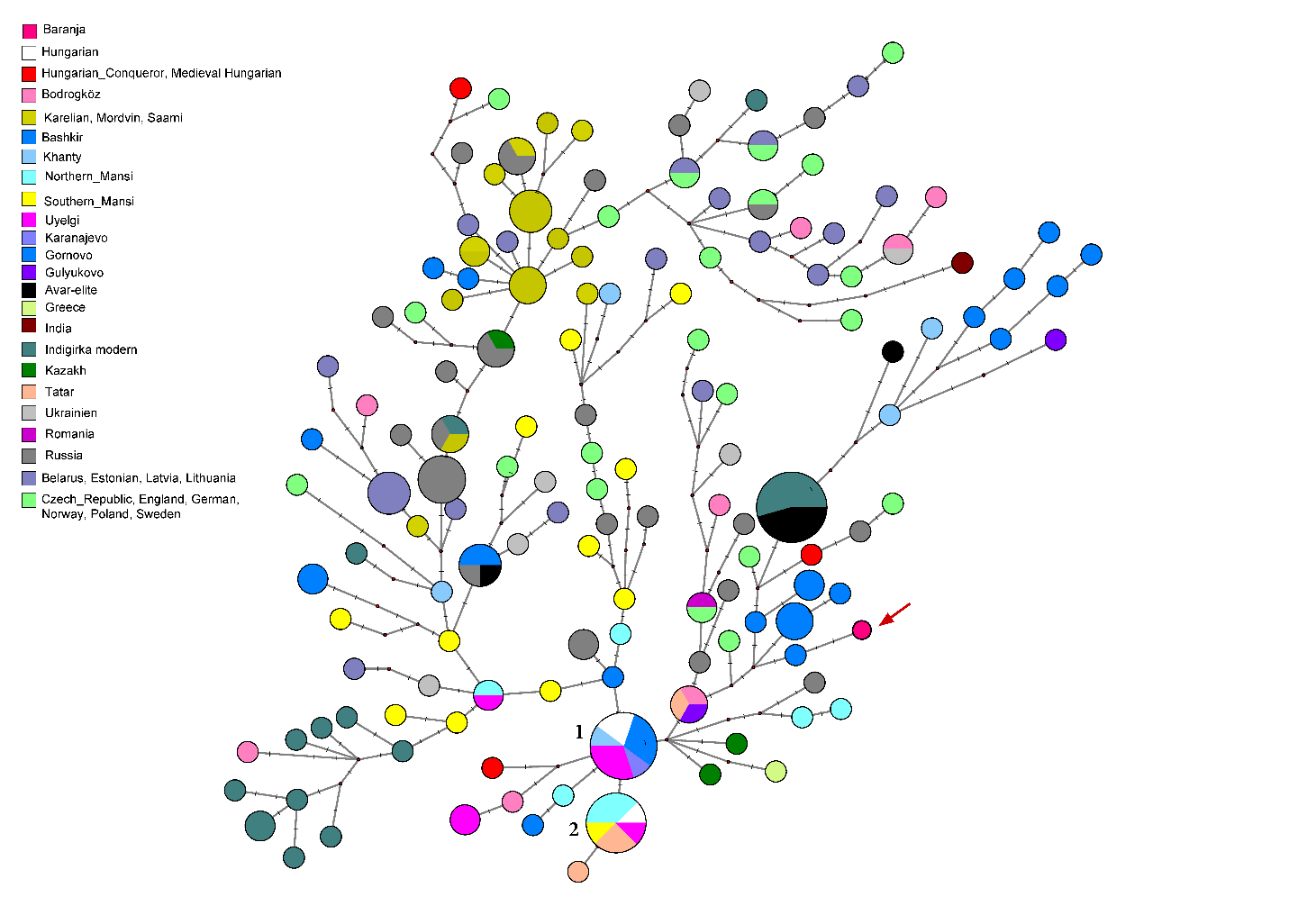


**Fig. S12 Median-Joining Network of 370 N-M46 haplotypes based on 15 STRs**

The circle sizes are proportional to the haplotype frequencies. The smallest area is equivalent to one individual.

**Mitochondrial DNA results**

**F_ST_ Analyses**

We analyzed the whole mitogenomes (16,569 base pairs) at the DNA sequence level and calculated Slatkin F_ST_ values (see Supplementary **Table S10**). A heatmap with clustering of F_ST_ values was created to visualize the genetic differentiation of the examined populations (**Fig. S13**). The Baranja and Zobor region populations cluster on the European branch with Hungarian-speakers, where the Czechs are the most similar to the Zobor region population.


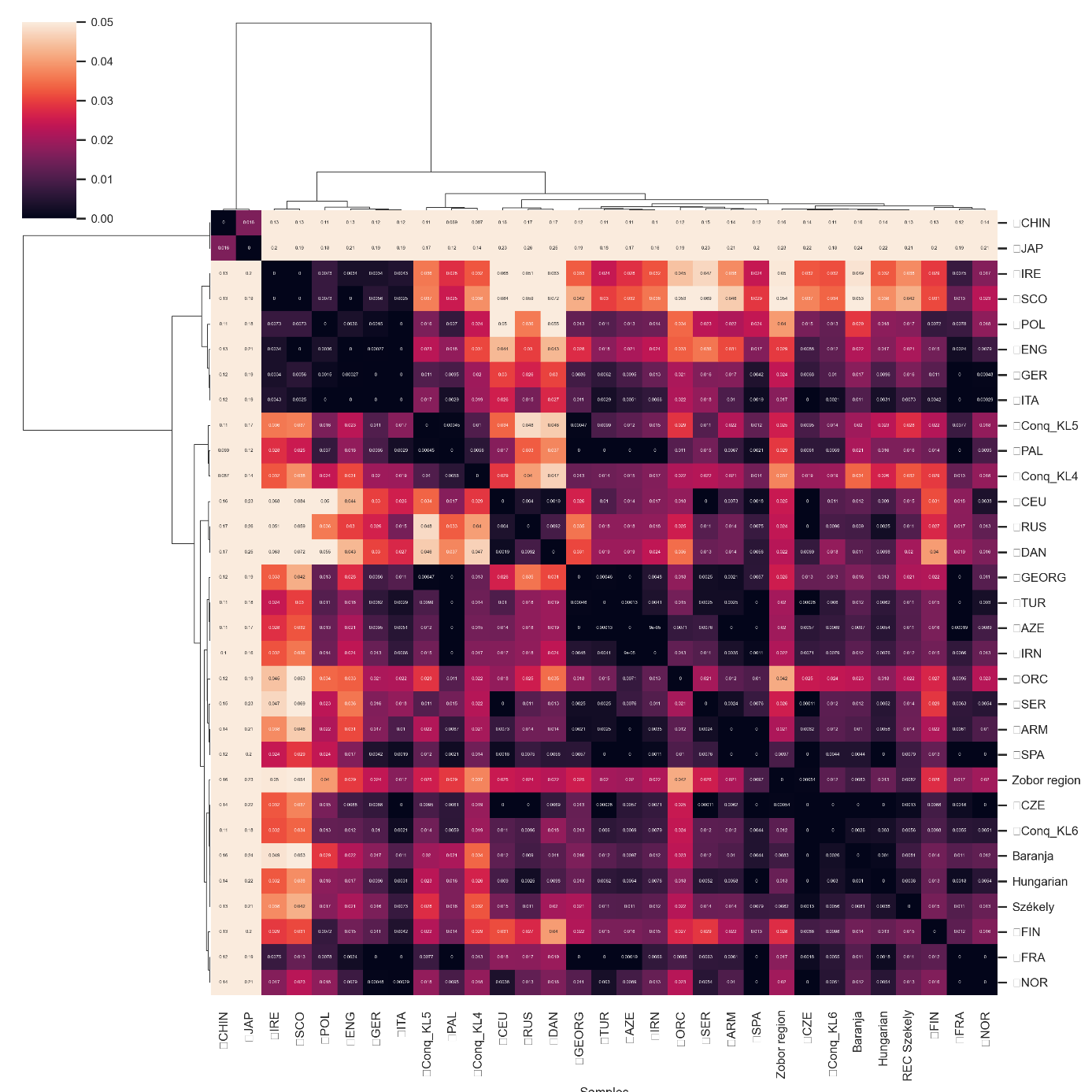


**Figure S13. Heatmap of pairwise F_ST_ values** (based on whole mitogenome sequences, see **Table S10**) for the investigated Baranja and Zobor region groups and 29 reference populations with a color scale ranging from yellow to dark purple. The lighter colors indicate larger genetic differentiation, whereas the darker colors show closer genetic affinities between the pairs of populations. The European groups all show great similarities with each other. We calculated the clustermap in Python using the seaborn clustermap function with parameters: metric = ‘correlation’, method = ‘complete’. Three conquest period populations were included in the dataset, as categorized by **Szeifert et al. (2022).**


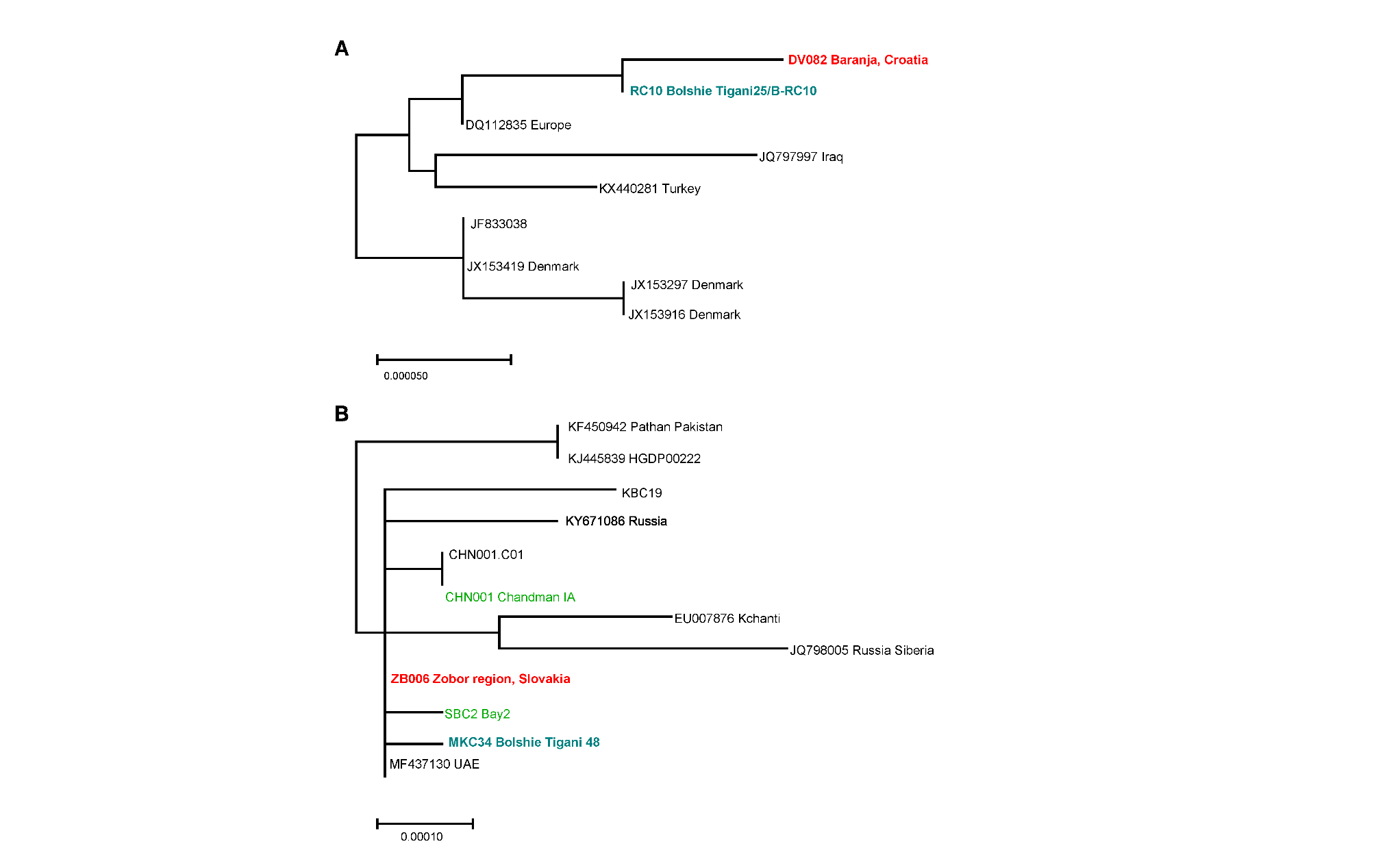


**Fig. S14 Parts of the neighbor-joining phylogenetic tree of mitochondrial haplogroup T1a**

The whole T1a tree was composed of 703 published mitogenomes.


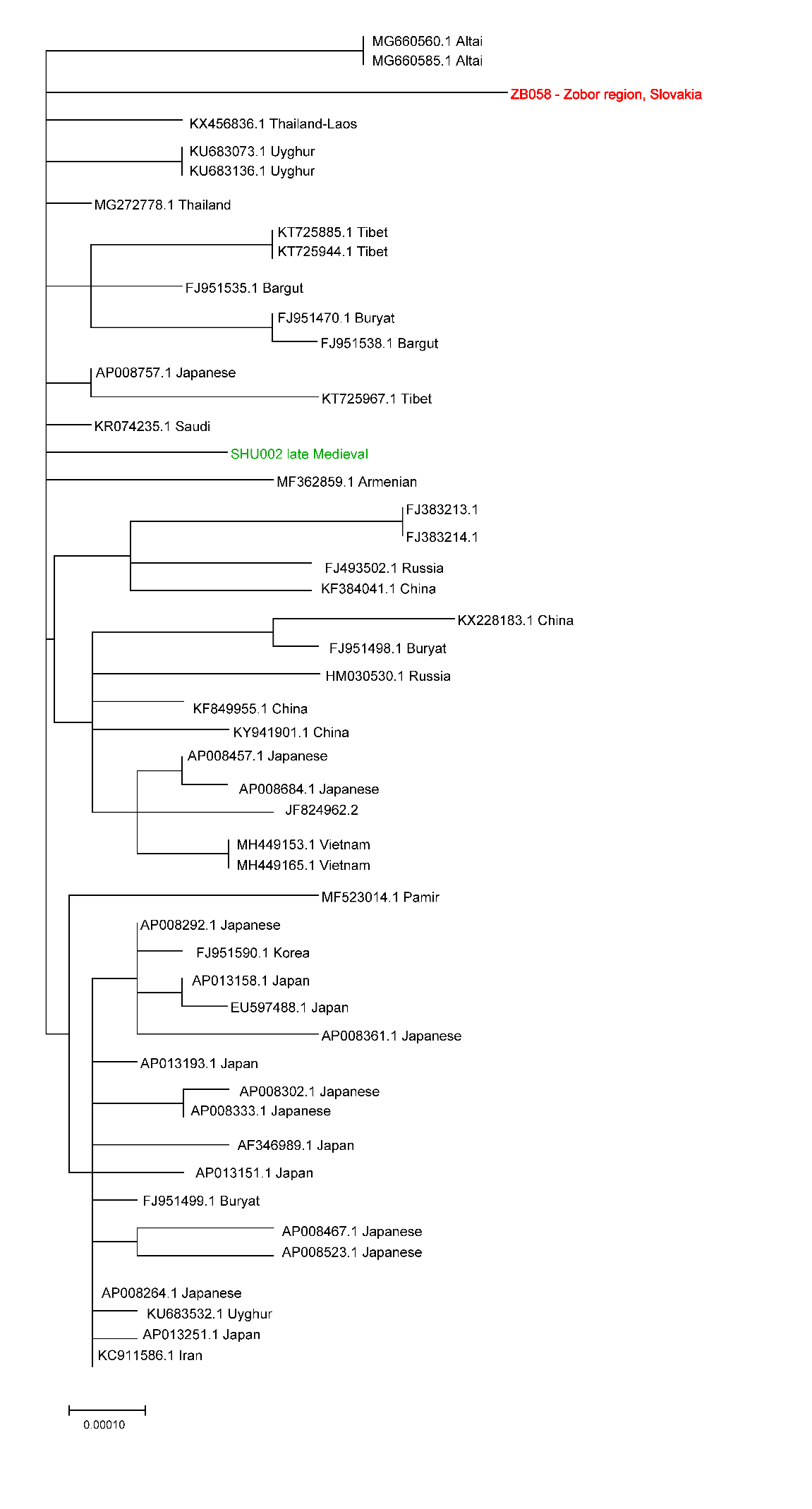


**Fig. S15 Part of the neighbor-joining phylogenetic tree of mitochondrial haplogroup D4b2.**

On the D4 neighbor-joining phylogenetic tree one sample from the Zobor region (D4b2b) falls close to modern-day Altaian, Armenian and Uyghur samples and to a late medieval period (SHU002) sample from Mongolia, Shunkhlai Uul ^50^.

Most of the data used for the mtDNA neighbor-joining networks are from the NCBI GenBank database, IDs and sources of other data are available in Supplementary **Table S11**. On the neighbor-joining phylogenetic trees the red color indicates the samples from the investigated Baranja and Zobor regions and modern-day Hungarian-speakers, sample names with light green color are ancient samples, teal color indicates ancient samples from the territory of today’s Hungary or historically Hungarian-related samples.

**Fig. S16 Neighbor-joining phylogenetic tree of mitochondrial haplogroup U2e**

**Fig. S17 Neighbor-joining phylogenetic tree of mitochondrial haplogroup U3**

**Fig. S18 Neighbor-joining phylogenetic tree of mitochondrial haplogroup U4**

**Fig. S19 Neighbor-joining phylogenetic tree of mitochondrial haplogroup U5a**

**Fig. S20 Neighbor-joining phylogenetic tree of mitochondrial haplogroup H13**

**Bibliography**

1. Lábadi, K. *Drávaszögi rovátkák*. (Horvátországi Magyarok Demokratikus Közössége, Eszék, 1993).

2. Katona, I. A drávaszögi folklór helye a néphagyományban. *A Janus Pannon. Múzeum Évkönyve* **38 (1993)**, 179–184 (1994).

3. Fügedi, E. *Nyitra megye betelepülése*. (Királyi Magyar Egyetemi Nyomda, 1938).

4. Kniezsa, I. *Adalékok a magyar-szlovák nyelvhatár történetéhez*. (Athenaeum, 1941).

5. Dávid, Z. *A magyar-szlovák nyelvhatár 1664-ben ez érsekujvári ejalet területén*. (KSH, 1997).

6. Tátrai, P. A Nyitrai járás etnikai földrajza. *Foldr. Ert.* **54**, 317–344 (2005).

7. Pamjav, H., Fóthi, Á., Fehér, T. & Fóthi, E. A study of the Bodrogköz population in north-eastern Hungary by Y chromosomal haplotypes and haplogroups. *Mol. Genet. Genomics* **292**, 883–894 (2017).

8. Pamjav, H. *et al.* The paternal genetic legacy of Hungarian-speaking Rétköz (Hungary) and Váh valley (Slovakia) populations. *Front. Genet.* **13**, 977517 (2022).

9. Borbély, N. *et al.* High Coverage Mitogenomes and Y-Chromosomal Typing Reveal Ancient Lineages in the Modern-Day Székely Population in Romania. *Genes (Basel).* **14**, (2023).

10. Bíró, A., Fehér, T., Bárány, G. & Pamjav, H. Testing Central and Inner Asian admixture among contemporary Hungarians. *Forensic Sci. Int. Genet.* **15**, 121–126 (2015).

11. Fehér, T. *et al.* Y-SNP L1034: limited genetic link between Mansi and Hungarian-speaking populations. *Mol. Genet. Genomics* **290**, 377–386 (2015).

12. Szeifert, B. *et al.* Tracing genetic connections of ancient Hungarians to the 6-14th century populations of the Volga-Ural region. *Hum. Mol. Genet.* **31**, 3266–3280 (2022).

13. Pimenoff, V. N. *et al.* Northwest Siberian Khanty and Mansi in the junction of West and East Eurasian gene pools as revealed by uniparental markers. *Eur. J. Hum. Genet.* **16**, 1254–1264 (2008).

14. Ilumäe, A. M. *et al.* Human Y Chromosome Haplogroup N: A Non-trivial Time-Resolved Phylogeography that Cuts across Language Families. *Am. J. Hum. Genet.* **99**, 163–173 (2016).

15. Fóthi, E. *et al.* Genetic analysis of male Hungarian Conquerors: European and Asian paternal lineages of the conquering Hungarian tribes. *Archaeol. Anthropol. Sci.* **12**, 31 (2020).

16. Karafet, T. M. *et al.* New binary polymorphisms reshape and increase resolution of the human Y chromosomal haplogroup tree. *Genome Res.* **18**, 830–838 (2008).

17. Rootsi, S. *et al.* A counter-clockwise northern route of the Y-chromosome haplogroup N from Southeast Asia towards Europe. *Eur. J. Hum. Genet.* **15**, 204–211 (2007).

18. Derenko, M. *et al.* Y-chromosome haplogroup N dispersals from south Siberia to Europe. *J. Hum. Genet.* **52**, 763–770 (2007).

19. Völgyi, A., Zalán, A., Szvetnik, E. & Pamjav, H. Hungarian population data for 11 Y-STR and 49 Y-SNP markers. *Forensic Sci. Int. Genet.* **3**, e27-8 (2009).

20. Neparáczki, E. *et al.* Y-chromosome haplogroups from Hun, Avar and conquering Hungarian period nomadic people of the Carpathian Basin. *Sci. Rep.* **9**, (2019).

21. Hallast, P. *et al.* The Y-chromosome tree bursts into leaf: 13,000 high-confidence SNPs covering the majority of known clades. *Mol. Biol. Evol.* **32**, 661–673 (2015).

22. Solé-Morata, N., Bertranpetit, J., Comas, D. & Calafell, F. Y-chromosome diversity in Catalan surname samples: insights into surname origin and frequency. *Eur. J. Hum. Genet.* **23**, 1549–1557 (2015).

23. FTDNA I2a Project. https://www.familytreedna.com/public/I2aHapGroup?iframe=ycolorized (2023).

24. Nováčková, J., Dreslerová, D., Černý, V. & Poloni, E. S. The place of Slovakian paternal diversity in the clinal European landscape. *Ann. Hum. Biol.* **42**, 511–522 (2015).

25. Rębała, K. *et al.* Contemporary paternal genetic landscape of Polish and German populations: from early medieval Slavic expansion to post-World War II resettlements. *Eur. J. Hum. Genet.* **21**, 415–422 (2013).

26. Pamjav, H., Fóthi, Fehér, T. & Fóthi, E. A study of the Bodrogköz population in north-eastern Hungary by Y chromosomal haplotypes and haplogroups. *Mol. Genet. Genomics* **292**, 883–894 (2017).

27. Semino, O. *et al.* The genetic legacy of Paleolithic Homo sapiens sapiens in extant Europeans: a Y chromosome perspective. *Science* **290**, 1155–1159 (2000).

28. Rootsi, S. *et al.* Phylogeography of Y-chromosome haplogroup I reveals distinct domains of prehistoric gene flow in Europe. *Am. J. Hum. Genet.* **75**, 128–137 (2004).

29. Peričić, M. *et al.* High-resolution phylogenetic analysis of southeastern Europe traces major episodes of paternal gene flow among slavic populations. *Mol. Biol. Evol.* **22**, 1964–1975 (2005).

30. Rootsi, S. *et al.* Distinguishing the co-ancestries of haplogroup G Y-chromosomes in the populations of Europe and the Caucasus. *Eur. J. Hum. Genet.* **20**, 1275–1282 (2012).

31. Berger, B. *et al.* High resolution mapping of y haplogroup G in Tyrol (Austria). *Forensic Sci. Int. Genet.* **7**, 529–536 (2013).

32. Nasidze, I. *et al.* Mitochondrial DNA and Y-chromosome variation in the caucasus. *Ann. Hum. Genet.* **68**, 205–221 (2004).

33. Phylotree. https://www.phylotree.org/. (2023).

34. Neparáczki, E. *et al.* Y-chromosome haplogroups from Hun, Avar and conquering Hungarian period nomadic people of the Carpathian Basin. *Sci. Rep.* **9**, 16569 (2019).

35. Lipson, M. *et al.* Parallel palaeogenomic transects reveal complex genetic history of early European farmers. *Nature* 114488 (2017) doi:10.1101/114488.

36. Dudás, E. *et al.* Genetic history of Bashkirian Mari and Southern Mansi ethnic groups in the Ural region. *Mol. Genet. Genomics* **294**, 919–930 (2019).

37. Yfull. www.yfull.com/tree (2023).

38. Haak, W. *et al.* Massive migration from the steppe was a source for Indo-European languages in Europe. *Nature* **522**, 207–211 (2015).

39. Rozhanskii, I. L. & Klyosov, A. A. Haplogroup R1a, Its Subclades and Branches in Europe During the Last 9,000 Years. *Adv. Anthropol.* **02**, 139–156 (2012).

40. Róna-Tas, A. *Hungarians and Europe in the early Middle Ages: an introduction to early Hungarian history*. (Central European University Press, 1999).

41. Battaglia, V. *et al.* Y-chromosomal evidence of the cultural diffusion of agriculture in Southeast Europe. *Eur. J. Hum. Genet.* **17**, 820–830 (2009).

42. Singh, S. *et al.* Dissecting the influence of Neolithic demic diffusion on Indian Y-chromosome pool through J2-M172 haplogroup. *Sci. Rep.* **6**, 19157 (2016).

43. Finocchio, A. *et al.* A finely resolved phylogeny of Y chromosome Hg J illuminates the processes of Phoenician and Greek colonizations in the Mediterranean. *Sci. Rep.* **8**, 7465 (2018).

44. Lazaridis, I. *et al.* A genetic probe into the ancient and medieval history of Southern Europe and West Asia. *Science (80-. ).* **377**, 940–951 (2022).

45. Wei, L.-H. *et al.* Whole-sequence analysis indicates that the Y chromosome C2*-Star Cluster traces back to ordinary Mongols, rather than Genghis Khan. *Eur. J. Hum. Genet.* **26**, 230–237 (2018).

46. Zerjal, T. *et al.* The genetic legacy of the Mongols. *Am. J. Hum. Genet.* **72**, 717–721 (2003).

47. Xue, Y. *et al.* Recent spread of a Y-chromosomal lineage in northern China and Mongolia. *Am. J. Hum. Genet.* **77**, 1112–1116 (2005).

48. Gnecchi-Ruscone, G. A. *et al.* Ancient genomic time transect from the Central Asian Steppe unravels the history of the Scythians. *Sci. Adv.* **7**, (2021).

49. Csáky, V. *et al.* Genetic insights into the social organisation of the Avar period elite in the 7th century AD Carpathian Basin. *Sci. Rep.* **10**, (2020).

50. Jeong, C. *et al.* A Dynamic 6,000-Year Genetic History of Eurasia’s Eastern Steppe. *Cell* **183**, 890-904.e29 (2020).
